# Neu1 deficiency and fibrotic lymph node microenvironment lead to imbalance in M1/M2 macrophage polarization

**DOI:** 10.3389/fimmu.2024.1462853

**Published:** 2024-09-13

**Authors:** Emilia Escalona, Alexandra Olate-Briones, Sofía Albornoz-Muñoz, Enzo Bonacic-Doric, Francisca Rodríguez-Arriaza, Andrés A. Herrada, Noelia Escobedo

**Affiliations:** Lymphatic Vasculature and Inflammation Research Laboratory, Instituto de Ciencias Biomédicas, Facultad de Ciencias de la Salud, Universidad Autónoma de Chile, Talca, Chile

**Keywords:** macrophage polarization, neuraminidase 1, fibrosis, TGF-β, lymph node microenvironment, sialidosis

## Abstract

Macrophages play a pivotal role in tissue homeostasis, pathogen defense, and inflammation resolution. M1 and M2 macrophage phenotypes represent two faces in a spectrum of responses to microenvironmental changes, crucial in both physiological and pathological conditions. Neuraminidase 1 (Neu1), a lysosomal and cell surface sialidase responsible for removing terminal sialic acid residues from glycoconjugates, modulates several macrophage functions, including phagocytosis and Toll-like receptor (TLR) signaling. Current evidence suggests that Neu1 expression influences M1/M2 macrophage phenotype alterations in the context of cardiovascular diseases, indicating a potential role for Neu1 in macrophage polarization. For this reason, we investigated the impact of Neu1 deficiency on macrophage polarization *in vitro* and *in vivo*. Using bone marrow-derived macrophages (BMDMs) and peritoneal macrophages from *Neu1* knockout (*Neu1^−/−^*) mice and wild-type (*WT*) littermate controls, we demonstrated that *Neu1*-deficient macrophages exhibit an aberrant M2-like phenotype, characterized by elevated macrophage mannose receptor 1 (MMR/CD206) expression and reduced responsiveness to M1 stimuli. This M2-like phenotype was also observed *in vivo* in peritoneal and splenic macrophages. However, lymph node (LN) macrophages from *Neu1^−/−^* mice exhibited phenotypic alterations with reduced CD206 expression. Further analysis revealed that peripheral LNs from *Neu1^−/−^* mice were highly fibrotic, with overexpression of transforming growth factor-beta 1 (TGF-β1) and hyperactivated TGF-β signaling in LN macrophages. Consistently, TGF-β1 was found to alter M1/M2 macrophage polarization *in vitro*. Our findings showed that *Neu1* deficiency prompts macrophages towards an M2 phenotype and that microenvironmental changes, particularly increased TGF-β1 in fibrotic tissues such as peripheral LNs in *Neu1^−/−^* mice, further influence M1/M2 macrophage polarization, highlighting its sensitivity to the local microenvironment. Therapeutic interventions targeting Neu1 or TGF-β signaling pathways may offer the potential to regulate macrophage behavior across different diseases.

## 1 Introduction

Macrophages are myeloid immune cells present in all mammalian tissues (1). Most tissue-resident macrophages arise from embryonic precursors that infiltrate tissues during prenatal stages, while the rest are derived from circulating precursors from the bone marrow (monocyte-derived macrophages), during inflammation or tissue repair (2, 3). Macrophages are one of the first lines of defense, acting as professional phagocytes and antigen-presenting cells under conditions of tissue infection, necrosis, or injury through the activation of pattern recognition receptors, such as Toll-like receptors (TLRs) or damage-associated molecular pattern receptors, among other receptors (4, 5). To initiate the immune response, macrophages respond to environmental stimuli, undergoing phenotypic changes to fulfill local requirements through a process known as macrophage polarization. Although macrophages exhibit wide heterogeneity and plasticity, they are typically referred to as classically activated macrophages (M1 phenotype) or alternatively activated macrophages (M2 phenotype), when they display an inflammatory state or play a role in tissue repair, respectively (6). Imbalance in macrophage polarization has significant impacts on the development of many pathologies, including cancer (7), cardiovascular diseases (8), autoimmune disorders, obesity, and inflammatory diseases (9). However, the mechanisms underlying macrophage polarization are still not fully understood.

Sialidases are enzymes that can selectively hydrolyze terminal sialic acid residues of glycoproteins or glycolipids (10, 11). To date, four types of mammalian sialidase isoforms, also known as neuraminidases, have been identified: Neuraminidase 1 (Neu1), Neu2, Neu3 and Neu4 (12). These neuraminidase isoforms differ in their cellular localization, chromosomal location, stability, optimal pH, and substrates (10). Among these, Neu1 is mainly found on lysosomal and cell surface membranes and plays a crucial role in macrophage function (11, 13). For instance, Neu1 is the major isoform contributing to sialidase activity and surface desialylation of monocytes during their differentiation into macrophages (13–16). Additionally, desialylation and Neu1 overexpression are linked to the secretion of pro-inflammatory cytokines in human monocytes (17, 18). Also, Neu1 is essential for macrophage phagocytosis, by removing sialic acid from the Fc gamma receptor 1 (FcγR1) and CD36 receptor (19, 20). Additionally, Neu1 modulates TLR-4 signaling, promoting the inflammatory response (21, 22). Recently, Wang et al. described that *Neu1*-deficient vascular macrophages exhibit an altered phenotype in an aortic dissection mouse model, switching to an anti-inflammatory phenotype (23). A similar finding was reported by Heimerl et al., where *Neu1*-deficient cardiac macrophages showed a less inflammatory phenotype in a model of ischemia/reperfusion injury (24). Although Neu1 has been shown to be upregulated in M1 and M2 macrophages (17), whether Neu1 has a direct effect on macrophage polarization has not yet been evaluated.

In this study, we tested whether *Neu1*-deficient macrophages could undergo normal polarization to M1 or M2 phenotypes. In addition, we studied the M1/M2 phenotype of resident macrophages in *Neu1^-/-^* mice and *WT* littermate controls. We found an abnormal M1/M2 phenotype *in vitro* in *Neu1-*deficient macrophages with main features of M2-like macrophages and increased M2-like macrophages in the spleen and the peritoneal cavity of *Neu1^-/-^* mice. Interestingly, M1 and M2 markers were reduced in macrophages from peripheral lymph nodes (pLNs) of *Neu1^-/-^* mice, which was associated with fibrosis and hyperactivated Transforming Growth Factor-β1 (TGF-β1) signaling. This microenvironment limited the response of *Neu1^-/-^* LN macrophages to inflammatory stimulus. Accordingly, TGF-β1 altered M1/M2 *in vitro* polarization. Our results suggest that the lack of Neu1 in macrophages skews these cells towards an M2 phenotype both *in vitro* and *in vivo*, but fibrosis and increased TGF-β signaling in pLNs of *Neu1^-/-^* mice further affect M1/M2 phenotype, decreasing pro- and anti-inflammatory macrophages, highlighting the importance of the microenvironment in modulating macrophage polarization.

## 2 Materials and methods

### 2.1 Animals

*Neu1^+/−^* mice used in this study were kindly gifted by Dr. Alessandra d’Azzo (St Jude Children´s Research Hospital, USA). Heterozygous males and females were crossed to generate null mice (*Neu1^-/-^*) and *Neu1^+/+^* littermate controls (herein *WT* mice). All animals were maintained on a C57Bl/6 background. Four to five-month-old mice were used in this study, and all mice were kept in a conventional animal facility with temperature- and light-controlled rooms, maintained on a 12-hour light/12-hour dark cycle, and received water and food *ad libitum.* All animal procedures and experiments were performed according to protocols approved by the Institutional Animal Care and Use Committee at Universidad Autónoma de Chile (protocol codes BE 05-21 and BS 04-21). Animals were maintained according to the “Guide to Care and Use of Experimental Animals, Canadian Council on Animal Care” and the study was conducted and reported in accordance with the ARRIVE Essential 10 guidelines.

### 2.2 Macrophages isolation, culture, and *in vitro* polarization

Bone marrow-derived macrophages (BMDMs) cultures were obtained as described previously (25, 26). Briefly, bone marrow cells were flushed from femurs and tibias with cold RPMI-1640 medium (HyClone, Utah, USA) and passed repeatedly through a 25-gauge needle until a homogenous suspension was observed. Red blood cells were lysed using Ammonium-Chloride-Potassium (ACK) lysing buffer (Gibco, NY, USA) for 3 minutes on ice. Cell viability was determined by trypan blue dye exclusion, and 2 × 10 ^5^ cells/well were seeded in 24-well plates in RPMI medium supplemented with 10% heat-inactivated fetal bovine serum (FBS, Corning, NY, USA), 100 U/ml penicillin-100 μg/ml streptomycin (HyClone), and 100 ng/ml macrophage colony-stimulating factor (M-CSF; Cat No. 576404, BioLegend, San Diego, CA, USA) and cultured at 37°C and 5% CO_2_. On day 4, an additional 100 ng/ml M-CSF was added. After 7 days, cultures contained >97% CD11b^+^F4/80^+^ macrophages as observed by FACS. Peritoneal macrophages (PMs) were obtained from the abdominal cavity of mice by repeated washing with 7 mL cold phosphate-buffered saline 1X (PBS) + FBS 3%, using a 21-G needle (27). Cell viability was evaluated by trypan blue dye exclusion, and cell characterization was performed by flow cytometer. Then, 8 x 10^5^ cells were seeded in 24-well plates to obtain macrophage-rich cultures, and peritoneal cells were incubated (37°C, 5% CO_2_) in RPMI alone for 90 minutes, washed extensively twice with pre-warmed RPMI, and the remaining adherent cells were incubated at 37°C and 5% CO_2_ in RPMI supplemented with 1% penicillin/streptomycin and 10% FBS. After 24 hours of culture, PMs were used for polarization assays. *In vitro* polarization of macrophages was performed by adding 100 ng/ml lipopolysaccharide (LPS, *E. Coli* O111:B4, Sigma-Aldrich, Saint Louis, USA), 100 ng/ml interferon-gamma (IFN-γ; Cat No. 575306, BioLegend) or 100 ng/ml LPS + 10 ng/ml IFN-γ to stimulate M1 polarization. For M2 polarization, we added 100 ng/ml interleukin-4 (IL-4; Cat No. 574304, BioLegend) or 20 ng/ml IL-4 + 10 ng/ml IL-13 (Cat No. 575902, BioLegend). Unstimulated macrophages were used as M0 macrophages (resting, control group). In some experiments, 20 ng/mL TGF-β1 (Cat No. 763102, BioLegend) or vehicle were added (28). M1 and M2 macrophage polarization was assessed by flow cytometry after 24 hours of BMDMs stimulation and after 24 or 48 hours of PMs polarization induction to analyze M1 and M2 macrophages, respectively. Cells were mechanically detached, washed, and resuspended in PBS + FBS 2%. M1 macrophages population was defined as live (7AAD^-^), F4/80^+^CD11b^+^CD86^+^CD206^-^, M2 macrophages were defined as F4/80^+^CD11b^+^CD86^-^CD206^+^, double positive macrophages (DP) were defined as F4/80^+^CD11b^+^CD86^+^CD206^+^ and double negative macrophages (DN) were defined as F4/80^+^CD11b^+^CD86^-^CD206^-^.

### 2.3 Flow cytometry

To detect macrophages, cells were stained with FITC-conjugated anti-F4/80 (Clone BM8.1, Cat No. 35-4801-U500, TONBO Biosciences, CA, USA) and Violet Fluor 500-conjugated anti-CD11b (Clone M1/70, Cat No. 85-0112-U100, TONBO biosciences). Additionally, APC/Cyanine7-conjugated anti-CD11c (Clone N418, Cat No. 117324, BioLegend) was used to exclude CD11c^high^ cells from secondary lymphoid organs (SLOs) analysis. For M1 macrophage staining, Brilliant Violet 421-conjugated anti-CD86 (Clone GL-1, Cat No. 105032, BioLegend) was used, and for M2 macrophage staining, Alexa Fluor-647-conjugated anti-CD206 (Clone C068C2, Cat No. 141712, BioLegend) and PE-conjugated anti-CD301 (Clone LOM-14, Cat No. 145704, BioLegend) were used. To study stromal cells, we stained with FITC-conjugated anti-mouse CD31 (Clone 390, Cat No. 102406, BioLegend), PE-conjugated anti-CD140A (PDGF Receptor α chain, Clone APA5, Cat No. 562776, BD Biosciences), PE/Cyanine7-conjugated anti-mouse podoplanin (Gp38, Clone 8.1.1, Cat No. 127412, BioLegend), APC/Cyanine7-conjugated anti-CD45 (Clone 30-F11, Cat No. 103116, BioLegend), Brilliant Violet 421-conjugated anti-mouse TER-119 (Cat No. 116233, BioLegend), and Brilliant Violet 510-conjugated anti-mouse Ly-6A/E (Sca-1) (Clone D7, Cat No. 108129, BioLegend). We also used Alexa Fluor-647-conjugated anti-Phospho-Smad2 (Ser465/Ser467) (Clone E8F3R, Cat No. 68550S, Cell Signaling Technology, MA, USA). For viability staining, we used 7-AAD Viability Staining Solution (Cat No. 420404, BioLegend) and 7-AAD-negative cells were gated as live cells. Samples were acquired on a FACSCanto II instrument and BD FACSDiva software (BD Biosciences, San Jose, CA, USA), and data were analyzed using FlowJo v10 (Tree Star, Inc.).

### 2.4 Tissue preparation to obtain SLO-derived cells

To obtain immune cells from spleens and pLNs, mice were anesthetized by inhalation of isoflurane (3%) in oxygen. After complete sedation, animals were perfused transcardially through the aorta with PBS using a peristaltic pump (Longer Pump Co., Ltd). pLNs and spleens were surgically removed and mechanically disaggregated in ice-cold RPMI. Fragmented tissues were passed through a 40-μm nylon mesh filter, and cells were centrifuged at 2000 rpm for 5 minutes. Additionally, splenocytes were incubated in ACK buffer (Gibco) for 5 minutes, centrifuged, and resuspended in ice-cold PBS/FBS 3%. To obtain stromal cells from pLNs, we adapted a previously described protocol (29). Briefly, after mechanically disrupting pLNs using a 25G needle in RPMI/FBS 2%, tissue fragments were incubated at 37°C and stirred for 30 minutes in digestion buffer 1 (1 mg/ml Collagenase IV, 40 μg/ml DNAse I in RPMI/FBS 2%). After centrifugation, supernatants were passed through a 70-μm nylon mesh filter, and cells were reserved. Next, pellets were digested in digestion buffer 2 (3.5 mg/ml Collagenase D, 40 μg/ml DNAse I in RPMI/FBS 2%) and incubated at 37°C in vigorous agitation with constant pipetting until a homogeneous suspension was achieved. To ensure a single-cell suspension, 5 mM EDTA was added. Finally, cells were passed through a 70-μm nylon mesh filter, centrifuged, and resuspended in PBS/FBS 2% for further analysis.

### 2.5 Trichrome masson staining

For the histopathological analysis, the samples were fixed in 4% paraformaldehyde in PBS for 24 hours. Later, samples were embedded in paraffin (Paraplast-Plus, Leica Biosystems) after being sequentially dehydrated using alcoholic solutions (ethanol 70%, ethanol 90%, ethanol 100%) and Neo-Clear™ Xylene Substitute (Sigma-Aldrich). Samples were sectioned to 10 µm thickness in a microtome (Biobase Biozone Co., China). For Trichrome Masson staining, sections were rehydrated by sequential incubation in Neo-Clear™ (twice) and alcoholic solutions (ethanol 100%, ethanol 95%, ethanol 70%, ethanol 50%) and stained with the Trichrome Stain Kit (Cat No. ab150686, Abcam, Cambridge, UK) according to the manufacturer’s instructions. Images were visualized using an Olympus BX51 microscope (Olympus America, Inc.).

### 2.6 Real-time PCR

Total RNA from BMDMs, spleens, and pLNs was isolated using TRIzol reagent according to the manufacturer’s instructions (Invitrogen, MA, USA). Reverse-transcription of 1 µg of RNA was performed using the High-Capacity RNA-to-cDNA Kit (Applied Biosystem, MA, USA) according to the manufacturer’s instructions. For the Real-time PCR, we used the Brilliant II SYBR Kit (Agilent Technologies, Santa Clara, CA, USA) and the primers listed in the **Table 1**. Each cDNA sample was analyzed in duplicate for quantitative assessment in the AriaMx Real-time PCR System (Agilent Technologies). Relative gene expression was calculated using the 2^-ΔΔCt^ method (30), and results were expressed relative to the housekeeping gene (*actin*).

**Table 1:** Primers used for quantitative RT-PCR analysis of mouse genes.

Gene	Sequence (5’-> 3’)	
*Mrc1 (CD206)*	Forward	GGTGGAAGAAGAAGTAGCCT
	Reverse	GAAGGGTCAGTCTGTGTTTG
*arg1*	Forward	AACACGGCAGTGGCTTTAACC
	Reverse	GGTTTTCATGTGGCGCATTC
*IL-1b*	Forward	GCAACTGTTCCTGAACTCAACT
	Reverse	ATCTTTTGGGGTCCGTCAACT
*IL-6*	Forward	CTGCAAGAGACTTCCATCCAGTT
	Reverse	GAAGTAGGGAAGGCCGTGG
*iNOS*	Forward	CGGAGCCTTTAGACCTCAACA
	Reverse	CCCTCGAAGGTGAGCTGAAC
*TGFB1*	Forward	GGAGAGCCCTGGATACCAACT
	Reverse	AGGACCTTGCTGTACTGTGTGT
*TGFB2*	Forward	GGCTTTCATTTGGCTTGAGATG
	Reverse	CTTCGGGTGAGACCACAAATAG
*COL1A1*	Forward	GCAAGAGGCGAGAGAGGTTT
	Reverse	GACCACGGGCACCATCTTTA
*COL3A1*	Forward	CTGTAACATGGAAACTGGGGAAA
	Reverse	CCATAGCTGAACTGAAAACCACC
*FN1*	Forward	GATGCCGATCAGAAGTTTGG
	Reverse	GGTTGTGCAGATCTCCTCGT
*actin*	Forward	GGCTGTATTCCCCTCCATCG
	Reverse	CCAGTTGGTAACAATGCCATGT

### 2.7 *In vivo* inflammation assay

To induce an *in vivo* inflammatory response, mice were intraperitoneally injected with PBS or LPS (1 mg/kg) as described before (31). The previously described Murine Sepsis Score (MSS) (32) was monitored at 1, 2, 3, 4, 6, 8, 10 and 24 hours without including the response to auditory stimuli. MSS was scored from 0 to 4 (higher scores indicating more severe symptoms), including assessments of appearance, level of consciousness, spontaneous activity, respiration rate and quality (labored breathing or gasping), and the aspect and secretion of the eyes. None of the mice reached a score of 4 for respiration rate and quality. The body weight of each mouse was recorded at the beginning and 24 hours after sepsis induction. After 24 hours post-injection, mice were euthanized, and spleens and peripheral lymph nodes (axillary and inguinal) were isolated and processed for flow cytometry.

### 2.8 Statistical analysis

Statistical analyses were performed using Prism software v8.0.1 (GraphPad Software Inc.). Data were expressed as mean ± standard error (SEM). Data were analyzed by unpaired *t*-test or using two-way ANOVA with Bonferroni *post-hoc* test. For statistical significance, we set the p-value < 0.05, expressed as *p ≤ 0.05, **p ≤ 0.01, and ***p ≤ 0.001.

## 3 Results

### 3.1 *Neu1*-deficient bone marrow-derived macrophages exhibit a predominant M2 phenotype under different polarization conditions

**Figure 1:**
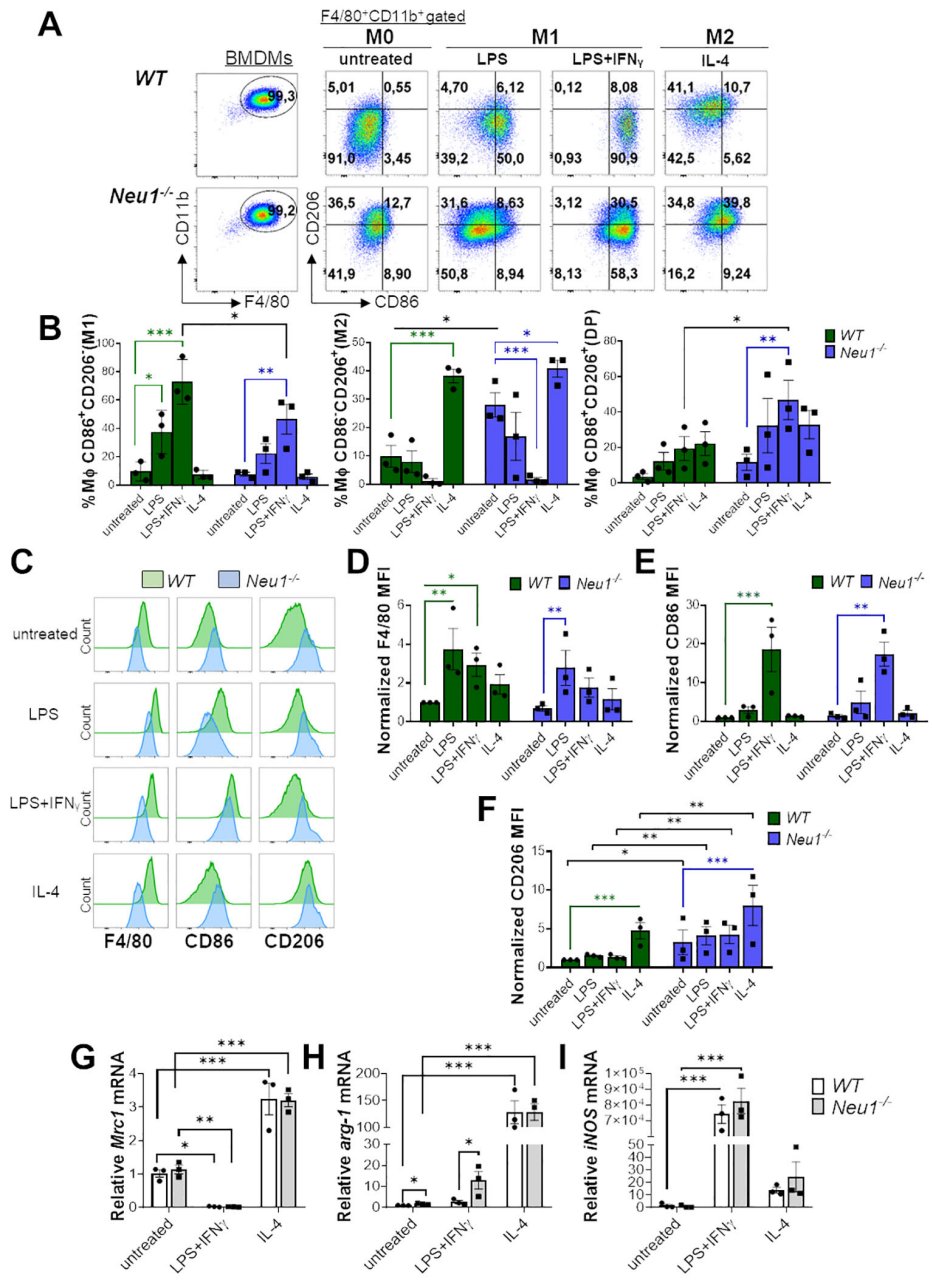
*Neu*1-deficient BMDMs overexpress the M2 marker CD206/MMR1 and exhibit an abnormal phenotype under *in vitro* M1/M2 polarization. **(A-F)** Analysis of *WT* and *Neu1* ^-/-^ BMDMs phenotypes after 24 hours of *in vitro* polarization. Macrophage differentiation to M1 was induced with LPS (100 ng/ml), and LPS plus IFN-γ (100 ng/ml and 10 ng/ml, respectively), while M2 macrophages were induced with IL-4 (100 ng/ml). As control we used unstimulated macrophages (M0 condition). **(A)** Representative pseudocolor dot-plots showing *WT* and *Neu1* ^-/-^ BMDMs (F4/80^+^CD11b^+^) (left), and MMR and CD86 expression in *WT* and *Neu1* ^-/-^ BMDMs after *in vitro* polarization (right). **(B)** Quantification of the frequencies of M1 (CD86^+^CD206^-^), M2 (CD86^-^CD206^+^) and double-positive (DP) (CD206^+^CD86^+^) macrophages in *WT* and *Neu1* ^-/-^ BMDMs after polarization. **(C-F)** Representative histograms and graphs of F4/80 **(D)**, CD86 **(E)** and CD206 **(F)** expression in *WT* (green) and *Neu1* ^-/-^ (blue) BMDMs after *in vitro* polarization. For quantification, mean fluorescence intensity (MFI) was normalized to the average of the untreated *WT* group. Bars represent mean ± SEM. *p < 0.05, **p < 0.01, ***p < 0.001 by two-way ANOVA with Bonferroni *post-hoc* test. Data from n=3 mice per group (*WT* and *Neu1* ^-/-^) across 3 independent experiments are shown. **(G-I)** Relative gene expression of mannose receptor C-type 1 (*Mrc-1*/CD206) **(G)**, arginase 1 (*arg-1*) **(H)** and inducible nitric oxide synthase (*iNOS*) **(I)** by Realtime-PCR in *WT* and *Neu1* ^-/-^ BMDMs after *in vitro* polarization. *Actin* was used as housekeeping. Data from n=3 were normalized to the average of the control *WT* group. Bars represent mean ± SEM. *p < 0.05, **p < 0.01, ***p < 0.001 by two-way ANOVA with Bonferroni *post-hoc* test. MΦ, macrophages.

Our initial approach to determine whether Neu1 plays a direct role in macrophage polarization was performing a classical *in vitro* polarization assay using BMDMs derived from *WT* and *Neu1^-/-^* mice. After 7 days of macrophage differentiation, cells were kept in medium alone (M0) or cultured under M1 differentiation conditions by using LPS alone, IFN-γ alone or LPS plus IFN-γ, while M2 differentiation was induced by culturing macrophages with IL-4 or IL-4 plus IL-13 (**Figure 1**, **Supplementary Figure S1**). To identify M1 and M2 macrophage populations, we used the previously described surface markers CD206 (also known as macrophage mannose receptor 1, MMR1) (M2) and CD86 (M1), respectively (33, 34). We observed increased levels of CD206 in BMDMs from *Neu1^-/-^* under M0 condition compared to *WT* BMDMs, suggesting a predominant M2 phenotype in resting *Neu1^-/-^* BMDMs (**Figures 1A, B, F**). Under different M1 stimuli, both *WT* and *Neu1^-/-^* BMDMs polarized to varying degrees to an M1 phenotype, as evidenced by the increase in the CD86^+^CD206^-^ macrophages percentage and the decrease in the CD86^-^CD206^+^ macrophages population (**Figures 1A, B**, **Supplementary Figures S1A, B**). It has been previously described that Neu1 removes sialic acid from the LPS receptor TLR-4, inducing receptor dimerization and decreasing TLR-4-Siglec E interaction, thereby promoting the activation of MyD88 and TRIF signaling during intracellular parasite infection (21, 22). When Neu1 expression or its membrane translocation is downregulated, LPS-TLR-4 signaling is interrupted (22), leading to a partial decrease in the proinflammatory response (35, 36). Consistently, we observed that LPS alone only polarized *WT* BMDMs to an M1 phenotype, but not *Neu1^-/-^* BMDMs (**Figure 1B**). Nevertheless, LPS induced the upregulation of F4/80 levels in *Neu1^-/-^* BMDMs, indicating that *Neu1^-/-^* BMDMs still respond to LPS (**Figures 1C, D**). Synergistic stimulation of LPS plus IFN-γ increased CD86 levels in both *WT* and *Neu1^-/-^* BMDMs (**Figures 1C, E**) compared to LPS (**Figures 1C, E**) or IFN-γ alone (**Supplementary Figures S1C, D**). In fact, the combination of LPS and IFN-γ was the most effective treatment to induce M1 macrophages (**Figure 1B**), but the percentage of M1 from *Neu1^-/-^* macrophages was significantly lower compared to *WT* BMDMs (**Figures 1A, B**). Additionally, we observed an elevated percentage of double-positive (DP) CD206^+^CD86^+^ macrophages in *Neu1^-/-^* BMDMs treated with LPS plus IFN-γ compared to *WT* BMDMs under the same treatment (**Figures 1A, B**). Under M2 treatments, both *WT* and *Neu1^-/-^* BMDMs significantly polarized to an M2 macrophage phenotype, evidenced by the increase in the CD86^-^CD206^+^ macrophages subpopulation (**Figures 1A, B**, **Supplementary Figures S1A, B**). However, CD206 expression was significantly higher in *Neu1^-/-^* BMDMs compared to *WT* BMDMs in both M2 conditions analyzed (**Figure 1F**, **Supplementary Figure S1D**), suggesting that the lack of *Neu1* expression in BMDMs impinges the cells to polarize to an M2 phenotype. To confirm these results, we evaluated M1/M2 gene expression in polarized BMDMs by RT-PCR analysis. Despite *CD206* gene expression being upregulated in M2 macrophages, data showed no significant changes between *Neu1^−/−^* and *WT* groups in any polarizing condition (**Figure 1G**), indicating that CD206 regulation by Neu1 likely occurs at post-translational level. Furthermore, we evaluated *arginase1* (*arg-1*) expression, a classical M2-associated gene (34). As previously reported, the expression of this gene is strongly induced after M2 stimulation (34). Consistent with our previous findings (**Figures 1A, B, F**), we found that *Neu1^-/-^* BMDMs expressed significantly more a*rg-1* under M0 and M1 conditions (**Figure 1H**), suggesting a stronger M2 polarization in *Neu1^-/-^* BMDMs compared to *WT* BMDMs. Regarding M1-associated genes expression, we showed that both *WT* and *Neu1^-/-^* BMDMs responded to M1 stimulation, strongly expressing inducible nitric oxide synthase (*iNOS*) (**Figure 1I**), supporting our FACS results. Overall, these results suggest that *Neu1^-/-^* BMDMs have a predominant M2 phenotype, responding more strongly to M2 stimuli and showing reduced M1 polarization under M1 conditions *in vitro*.

### 3.2 Neu1 deficiency alters peritoneal macrophages polarization towards a M2 phenotype

**Figure 2:**
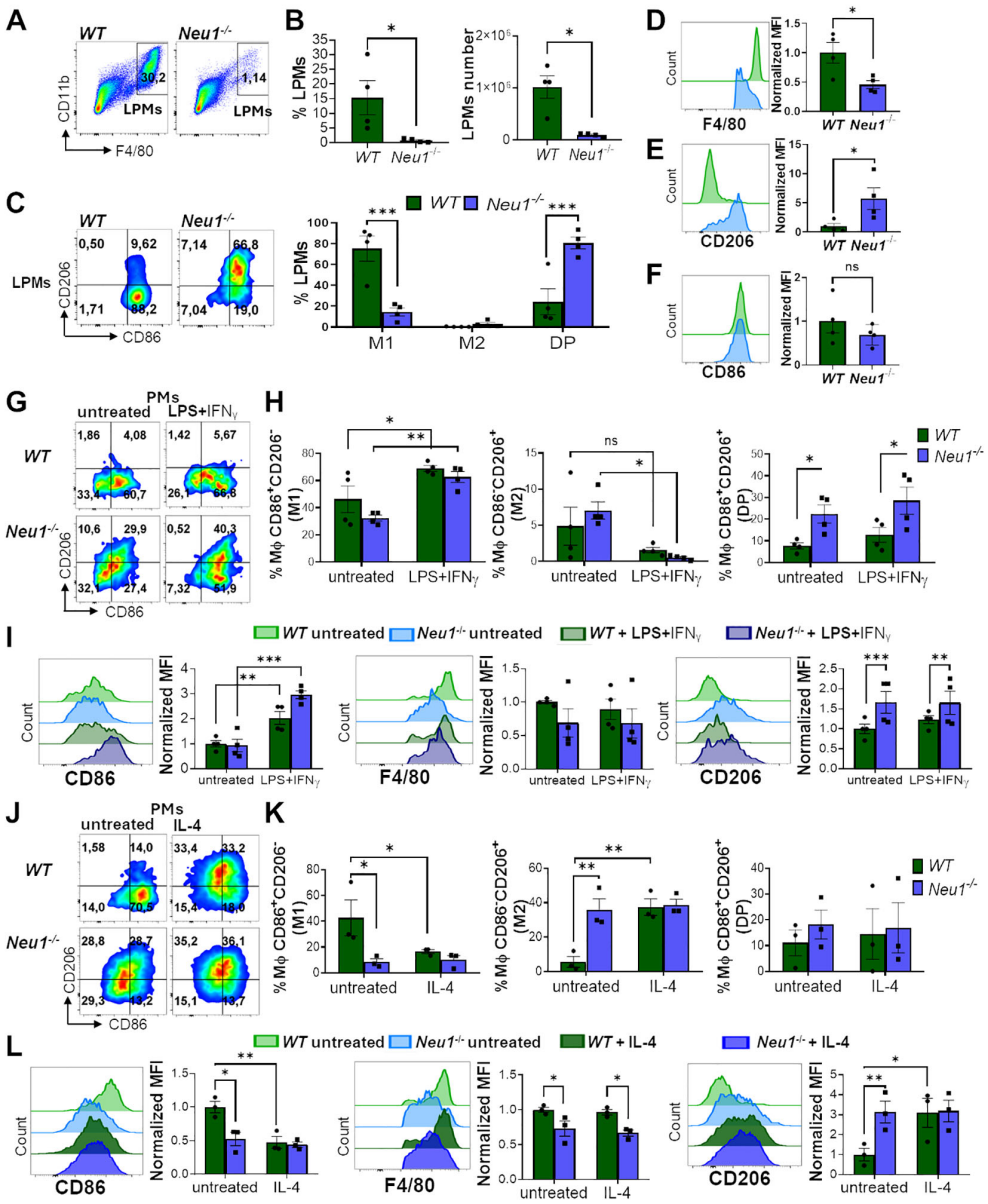
Peritoneal macrophages (PMs) of *Neu1^-/-^* mice display M2 phenotype and alterations in the *in vitro* polarization assay. **(A-F)** Analysis of large peritoneal macrophages (LPMs) from *WT* and *Neu1* ^-/-^ mice. **(A)** Representative pseudocolor dot-plots showing LPMs (CD11b^+^F4/80^high^). **(B)** Quantification of the frequency and number of LPMs in *WT* and *Neu1^-/-^* mice. Bars represent mean ± SEM. *p < 0.05 by unpaired *t*-test. **(C)** Representative pseudocolor dot-plots displaying CD206 and CD86 expression in LPMs of *WT* and *Neu1^-/-^* mice (left) and quantification of M1, M2 and double-positive (DP) LPMs frequencies (right). Bars represent mean ± SEM. *p < 0.05 by unpaired *t*-test. **(D-F)** Representative histograms and graphs of F4/80 **(D)**, CD206 **(E)** and CD86 **(F)** expression in *WT* (green) and *Neu*1^-/-^ (blue) LPMs. For quantification, mean fluorescence intensity (MFI) was normalized relative to the average of *WT* mice. Bars represent mean ± SEM. *p < 0.05, ***p < 0.001 by two-way ANOVA with Bonferroni *post-hoc* test. Data from 4 mice per group are shown. **(G-I)** Assessment of *WT* and *Neu1^-/-^* PMs phenotype after M1 *in vitro* polarization. **(G)** Representative pseudocolor dot-plots showing CD206 and CD86 expression in *WT* and *Neu1* ^-/-^PMs after 24 hours of M1 *in vitro* polarization with LPS plus IFN-γ (100ng/ml and 10ng/ml, respectively). **(H)** Quantification of M1, M2 and DP frequencies of *WT* and *Neu1* ^-/-^ PMs after M1 polarization. **(I)** Representative histograms and MFI graphs of CD86, F4/80 and CD206 in *WT* and *Neu1* ^-/-^ PMs after M1 polarization. Bars represent mean ± SEM. ns: not significant, *p < 0.05, **p < 0.01, ***p < 0.001 by two-way ANOVA with Bonferroni *post-hoc* test. Data from n=4 from 3 independent experiments. **(J-L)** Assessment of *WT* and *Neu1^-/-^* PMs phenotype after M2 *in vitro* polarization. **(J)** Representative pseudocolor dot-plots showing CD206 and CD86 expression in *WT* and *Neu1* ^-/-^ PMs after 48 hours of M2 *in vitro* polarization with 100 ng/ml of IL-4. **(K)** Quantification of M1, M2 and DP frequencies of *WT* and *Neu1* ^-/-^ PMs after M2 polarization. **(L)** Representative histograms and graphs of CD86, F4/80 and CD206 expression in *WT* and *Neu1* ^-/-^ PMs after M2 polarization. MFI were normalized relative to the average of the untreated *WT* group. Bars represent mean ± SEM. *p < 0.05, **p < 0.01 by two-way ANOVA with Bonferroni *post-hoc* test. Data from n=3 from 3 independent experiments. MΦ, macrophages.

To better understand the effect of Neu1 deficiency on macrophage polarization, we also analyzed PMs from *WT* and *Neu1^-/-^* mice. Based on CD11b and F4/80 expression, we analyzed large PMs (LPMs) (F4/80^high^CD11b^+^), isolated from the peritoneal cavity of mice (37, 38). Although we attempted to analyze small peritoneal macrophages by gating the CD11b^+^F4/80^low^ population, their minimal presence in *Neu1^-/-^* mice limited the quantification. First, we observed a drastic reduction in the percentage and number of LPMs recovered from the peritoneal fluid of *Neu1* ^-/-^ mice compared to their littermate controls (**Figures 2A, B**). *Neu1^-/-^* LPMs were significantly more double-positive (DP) (CD86^+^CD206^+^) and less M1 (CD86^+^CD206^-^) than *WT* LPMs (**Figure 2C**). Additionally, *Neu1^-/-^* LPMs showed significantly lower F4/80 (**Figure 2D**) and higher CD206 (**Figure 2E**) levels compared to *WT* LPMs, indicating a strong M2 phenotype, similar to what we observed in *Neu1^-/-^* BMDMs *in vitro*. However, *Neu1^-/-^* LPMs showed no significant differences in CD86 (**Figure 2F**), which explains the increase in the DP phenotype in *Neu1^-/-^* LPMs (**Figure 2C**). Next, we evaluated the ability of *WT* and *Neu1^-/-^* PMs to polarize *in vitro*. Under M1 polarization condition, both *WT* and *Neu1^-/-^* PMs acquired an M1 phenotype (CD86^+^CD206^-^) (**Figures 2G, H**) and increased CD86 levels (**Figure 2I**), demonstrating the capacity of *Neu1^-/-^* PMs to respond to inflammatory stimuli. Nevertheless, M1 *Neu1^-/-^* PMs maintained high CD206 expression (**Figure 2I**) and an elevated DP (CD86^+^CD206^+^) phenotype (**Figures 2G, H**), revealing an abnormal M1 polarization, as observed in *Neu1^-/-^* BMDMs. Under M2 polarization conditions, *WT* PMs were capable of acquiring an M2 phenotype by significantly decreasing CD86^+^CD206^-^ (M1) macrophages and CD86 expression, while significantly increasing the CD86^-^CD206^+^ (M2) population and CD206 expression (**Figures 2J–L**). In contrast, *Neu1^-/-^* PMs did not further increase the M2 phenotype and CD206 expression or reduce the M1 phenotype and CD86 expression, suggesting that the M2 phenotype in this cell population had reached a plateau (**Figures 2J–L**). In summary, *Neu1^-/-^* PMs displayed an M2 macrophage phenotype under homeostatic condition, which reduced their responsiveness to M2 stimuli but could be modulated by M1 polarization. In line with our observations in BMDMs, these findings suggest that Neu1 deficiency leads to an increased M2 phenotype, indicating dysregulation in their response to polarization cues.

### 3.3 Secondary lymphoid organs-associated macrophages in *Neu1^-/-^* mice exhibited phenotypic alterations

**Figure 3:**
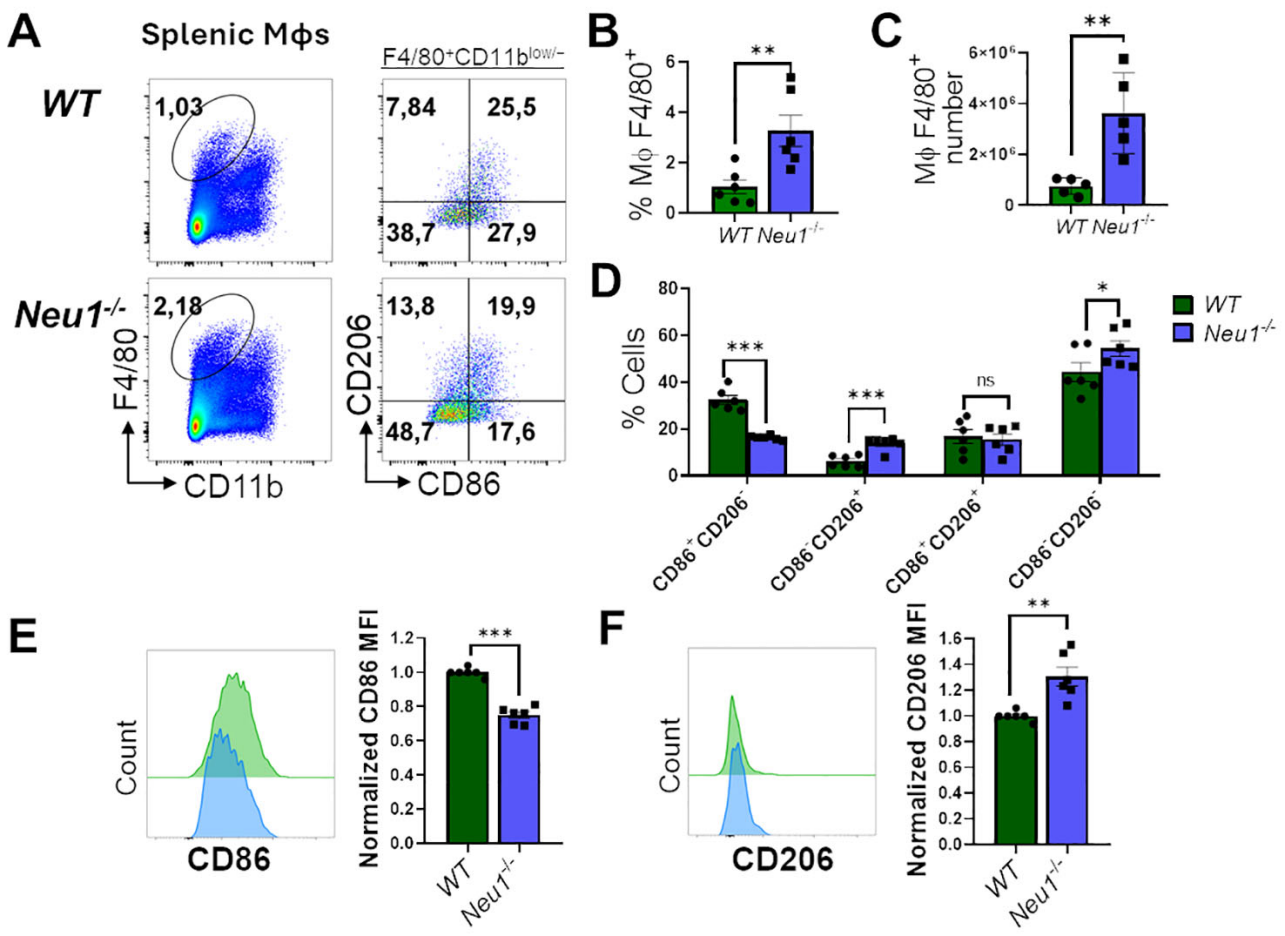
*Neu*1^-/-^ splenic macrophages show an aberrant M1/M2 phenotype. **(A)** Representative pseudocolor dot-plots of splenic macrophages (F4/80^+^, CD11b^low/−^) (left) and CD206 and CD86 expression (right). **(B, C)** Quantification of frequency **(B)** and number **(C)** of F4/80^+^ splenic macrophages from *WT* and *Neu*1^-/-^ mice. **(D)** Quantification of CD86^+^CD206^-^, CD86^-^CD206^+^, double-positive (DP) (CD206^+^CD86^+^) and double-negative (DN) (CD206^-^CD86^-^) frequencies of *WT* and *Neu1* ^-/-^ splenic macrophages. **(E, F)** Representative histograms and relative expression of CD86 **(E)** and CD206 **(F)** in *WT* (green) and *Neu1* ^-/-^ (blue) splenic macrophages. Mean fluorescence intensity (MFI) was normalized relative to the average of *WT* mice. Bars represent mean ± SEM. ns: not significant, *p < 0.05, **p < 0.01, ***p < 0.001 by unpaired *t*-test for **(B, C, E, F)** and two-way ANOVA with Bonferroni *post-hoc* test for **(D)** Data from n=6 from 5 independent experiments. MΦ, macrophages.

The adaptive immune response occurs in SLOs, where resident macrophages play a crucial role in tissue homeostasis and immune response (39, 40). Therefore, we investigated whether macrophages associated with SLOs from *Neu1^-/-^* mice may also have phenotypic alterations. First, we examined M1/M2 markers in splenic macrophages from *Neu1*-deficient mice and their littermate controls. It has been reported that resident splenic macrophages are F4/80^+^CD11b^low/−^ (41); hence, we focused on analyzing F4/80^+^CD11b^low/−^ macrophages derived from spleens of *WT* and *Neu1^-/-^* mice. Contrary to our results in the peritoneal cavity, we found a significantly higher percentage (**Figures 3A, B**) and number (**Figure 3C**) of F4/80^+^ splenic macrophages in *Neu1^-/-^* mice compared to *WT* mice. However, in concordance with our previous findings, *Neu1*-deficient splenic macrophages were significantly less CD86^+^CD206^-^ and more CD86^-^CD206^+^ (**Figure 3D**). This was associated with the overall downregulation of CD86 (**Figure 3E**) and an upregulation of CD206 (**Figure 3F**). In summary, F4/80^+^ splenic macrophages from *Neu1^-/-^* mice have an aberrant phenotype, showing mainly M2-like features.

**Figure 4:**
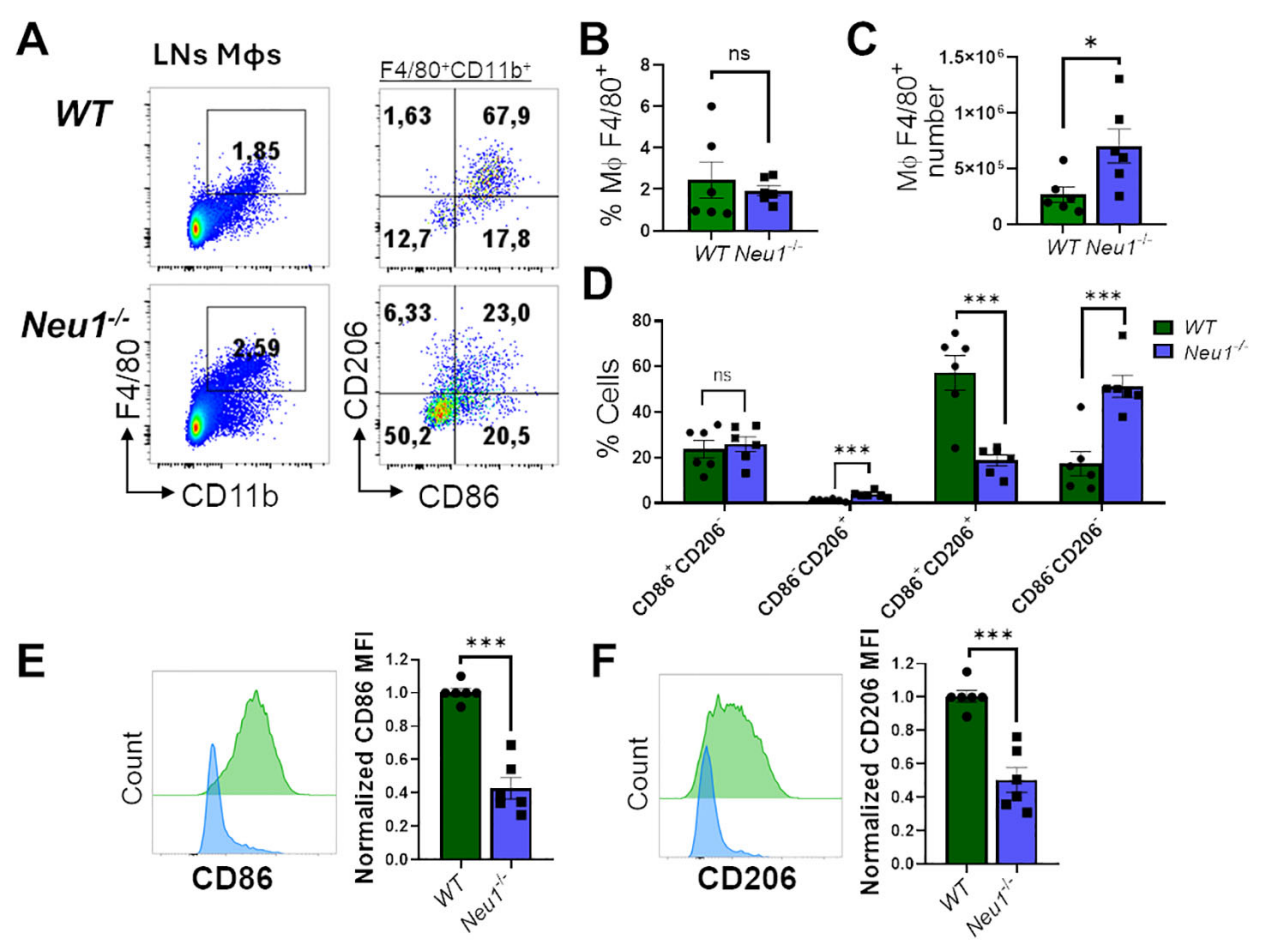
Lymph nodes (LN) macrophages of *Neu*1^-/-^ mice are predominantly double negative (CD86^-^CD206^-^) and have diminished M1 and M2 markers. **(A)** Representative pseudocolor dot-plots of LN macrophages (CD11b^+^ F4/80^+^) (left) and their CD206 and CD86 expression (right). **(B, C)** Quantification of frequency **(B)** and number **(C)** of pLNs from *WT* and *Neu*1^-/-^ mice. **(D)** Quantification of CD86^+^CD206^-^, CD86^-^CD206^+^, double-positive (DP) (CD206^+^CD86^+^) and double-negative (DN) (CD206^-^CD86^-^) frequencies of LN macrophages. **(E, F)**. Representative histograms and relative expression of CD86 **(E)** and CD206 **(F)** in LN macrophages from *WT* (green) and *Neu*1^-/-^ (blue) mice. Mean fluorescence intensity (MFI) was normalized relative to the average of *WT* mice. Bars represent mean ± SEM. ns: not significant, *p < 0.05, ***p < 0.001 by unpaired *t*-test for **(B, C, E, F)** and two-way ANOVA with Bonferroni *post-hoc* test for **(D)** Data from n=6 from 5 independent experiments. MΦ, macrophages.

We then analyzed M1/M2 markers in F4/80^+^CD11b^+^ macrophages from peripheral lymph nodes (pLNs) of *WT* and *Neu1^-/-^* mice. *Neu1^-/-^* mice had more macrophages in their pLNs compared to littermate controls, although the macrophage frequency was not significantly different (**Figures 4A–C**). Regarding M1/M2 phenotype distribution, most F4/80^+^ lymph node (LN) macrophages from *WT* mice exhibited an intermediate DP phenotype (CD206^+^CD86^+^), whereas F4/80^+^ LN macrophages from *Neu1^-/-^* mice showed an increased CD86^-^CD206^+^ population, although the DN population (CD86^-^CD206^-^) was the most predominantly expanded population (**Figure 4D**). *Neu1^-/-^* F4/80^+^CD11b^+^ LN macrophages, similar to *Neu1^-/-^* F4/80^+^ CD11b^low/−^ splenic macrophages, also expressed significantly less CD86 (**Figure 4E**). However, LN macrophages from *Neu1^-/-^* mice downregulated CD206 levels compared to LN macrophages from *WT* animals (**Figure 4F**), consistent with the increase in the DN population observed in pLNs from *Neu1^-/-^* mice. Overall, these results suggest that, aside from the abnormal phenotype in *Neu1^-/-^* splenic and LN macrophages, microenvironment factors in the pLNs of *Neu1^-/-^* mice could be contributing to the expansion of the DN macrophage population particularly observed in this tissue.

### 3.4 pLNs from *Neu1^-/-^* mice have fibrosis, increased TGF-β1 expression and TGF-β-responding macrophages

**Figure 5:**
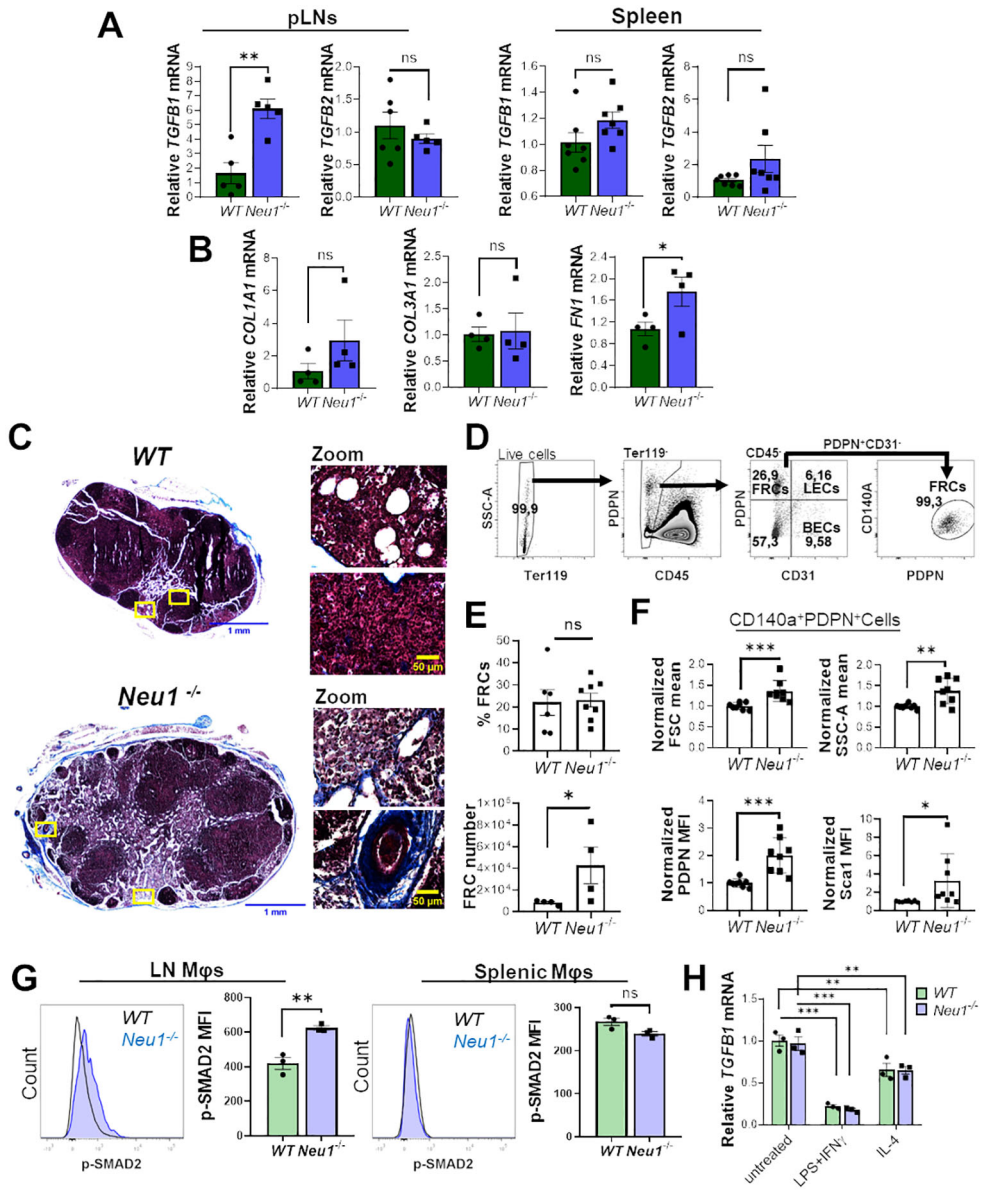
Peripheral lymph nodes (pLNs) of *Neu*1^-/-^ mice have increased fibrosis and activation of TGF-β signaling in lymph node macrophages. **(A)** Relative expression of *TGFB1* and *TGFB2* in pLNs and spleens of *WT* and *Neu*1^-/-^ mice. *Actin* was used as housekeeping. Bars show the mean ± SEM. Data from at least n=5 of 3 independent experiments were analyzed by unpaired *t*-test. ns: not significant, **p < 0.01. **(B)** Relative expression of collagen type I alpha 1 chain (*COL1A1*), collagen type III alpha 1 chain (*COL3A1*) and fibronectin (*FN1*) in pLNs of *WT* and *Neu*1^-/-^ mice. *Actin* was used as housekeeping. Bars show the mean ± SEM, Data n=4. from 2 independent experiments were analyzed by unpaired *t*-test. ns: not significant; *p < 0.05. **(C)** Representative images of Masson’s trichrome staining of inguinal LN sections of *WT* and *Neu*1^-/-^ mice, scale bar represent 1 mm in whole LN section (left) and 50 µM in zooms (right). Data from 2 independent experiments is shown. **(D-F)** Analysis of fibroblastic reticular cells (FRCs) of pLNs of *WT* and *Neu1^-/-^* mice. **(D)** Flow cytometry gating strategy to select FRCs (Ter119^-^CD45^-^CD31^-^CD140a^+^PDPN^+^ cells). **(E)** Quantification of the frequency and cells number of FRCs in pLNs of *WT* and *Neu*1^-/-^ mice. Bars show mean ± SEM. Data from at least n=4 from 3 independent experiments were analyzed by unpaired *t* test, ns: not significant, *p < 0.05. **(F)** Graphs of FSC mean, SSC-A mean, podoplanin (PDPN) and Sca1 expression in FRCs of pLNs of *WT* and *Neu*1^-/-^ mice. Mean fluorescence intensity (MFI) was normalized relative to the average of *WT* mice. Bars show mean ± SEM (n=8) from 3 independent experiments and were analyzed by unpaired *t* test, *p < 0.05, **p < 0.01, ***p < 0.001. **(G)** Representative histograms and MFI quantification of pSMAD-2 in F4/80^+^ LN and splenic macrophages of *WT* and *Neu*1^-/-^ mice. Bars show mean ± SEM (n=3). Data were analyzed by unpaired *t* test. ns: not significant, **p < 0.01. **(H)** Relative expression of *TGFB1* in *WT* and *Neu*1^-/-^ BMDMs after 24 hours of culture under different conditions: M0 (untreated), M1 (100 ng/ml LPS plus 10 ng/ml IFN-γ) or M2 (100 ng/ml IL-4). *Actin* was used as housekeeping gene. Data from n=3 were normalized to the average of the control *WT* group. Bars represent mean ± SEM. **p < 0.01, ***p < 0.001 by two-way ANOVA with Bonferroni *post-hoc* test. MΦ, macrophages.

It was previously reported that *Neu1^-/-^* mice exhibit fibrosis in several organs associated with the upregulation of TGF-β (42). Since TGF-β is an immunomodulatory cytokine (43), we hypothesized that it could be involved in the expansion of DN macrophages observed in the lymph node of *Neu1^-/-^* mice. To test this hypothesis, we first evaluated the expression of TGF-β1 and TGF-β2 in the SLOs of both mice genotypes. We only found significantly higher expression of *TGFB1* isoform in pLNs of *Neu1^-/-^* mice compared to *WT* animals, but not in the spleen (**Figure 5A**), supporting the idea that TGF-β1 could be involved in the altered phenotype of macrophages in the pLNs of *Neu1^-/-^* mice. Additionally, we evaluated the expression of other fibrotic-associated genes in the pLNs. *Neu1^-/-^* mice showed significantly higher expression of fibronectin (*FN1*), but not in collagen type I alpha 1 chain (*COL1A1*) and collagen type III alpha 1 chain (*COL3A1*), compared to littermate controls (**Figure 5B**). Masson’s trichrome staining confirmed fibrosis in the LN of *Neu1^-/-^* mice and revealed architectural changes (**Figure 5C**), indicating an abnormal immunological microenvironment. To better characterize the fibrotic status of pLNs in *Neu1^-/-^* mice, we analyzed the LN fibroblastic reticular cells (FRCs) identifying them as Ter119^-^CD45^-^CD31^-^PDPN^+^CD140a^+^ by flow cytometry (**Figure 5D**) (44). Although we did not find significant differences in the percentage of fibroblasts between the two groups, pLNs of *Neu1^-/-^* mice exhibited higher number of FRCs (**Figure 5E**). Additionally, we found that FRCs from pLNs from *Neu1^-/-^* mice had an altered phenotype with increased size and complexity (**Figure 5F**), reflecting an activated status. Moreover, FRCs from pLNs of *Neu1^-/-^* mice showed elevated levels of podoplanin (PDPN), crucial for controlling adhesion, elongation, and contractility of activated fibroblasts in LN microarchitecture (45) (**Figure 5F**). Interestingly, FRCs of pLNs from *Neu1^-/-^* mice also exhibit elevated levels of stem cells antigen 1 (Sca1) (**Figure 5F**), a protein associated with stem features in fibroblast (46), suggesting that FRCs from pLNs of *Neu1^-/-^* mice may possess a transitional epithelial/mesenchymal status, consistent with what was previously reported in *Neu1^-/-^* fibroblast (42). These phenotypic alterations suggest that FRCs may contribute to tissue disturbance in pLNs of *Neu1^-/-^* mice. Altogether, these findings evidenced that pLNs of *Neu1^-/-^* mice are highly fibrotic, along with having increased levels of TGF-β1. Then, to evaluate the response of macrophages to TGF-β in pLNs, we assessed the status of TGF-β signaling in LN macrophages. The activation of TGF-β receptors leads to the phosphorylation of the transcription factor SMAD2 (47). Therefore, we examined pSMAD2 levels as an indicator of the activation of TGF-β signaling in this cell population. Higher levels of p-SMAD2 were observed in LN macrophages from *Neu1^-/-^* mice compared to their littermate controls (**Figure 5G**), suggesting that this population responds to the increased TGF-β1 levels in this microenvironment. In contrast, splenic macrophages did not show upregulation of p-SMAD2 (**Figure 5G**). Finally, since macrophages are capable to produce TGF-β (48, 49), we assessed TGF-β1 expression in *WT* or *Neu1* ^-/-^ BMDMs under M0, M1 and M2 conditions. We observed that, while the different polarizing conditions influenced TGF-β1 expression as previously described (48), no significant differences of TGF-β1 levels were detected between *WT* and *Neu1* ^-/-^ macrophages in any condition (**Figure 5H**), suggesting that Neu1 deficiency does not affect TGF-β1 expression in macrophages. Overall, these results indicate that LN macrophages from *Neu1^-/-^* mice have hyperactivated TGF-β signaling, strongly suggesting that TGF-β1, and possibly FRCs may play a significant role in contributing to the altered M1/M2 phenotype observed in this tissue.

### 3.5 TGF-β1 modulates M1 and M2 macrophage polarization in *WT* and *Neu1^-/-^* macrophages

**Figure 6:**
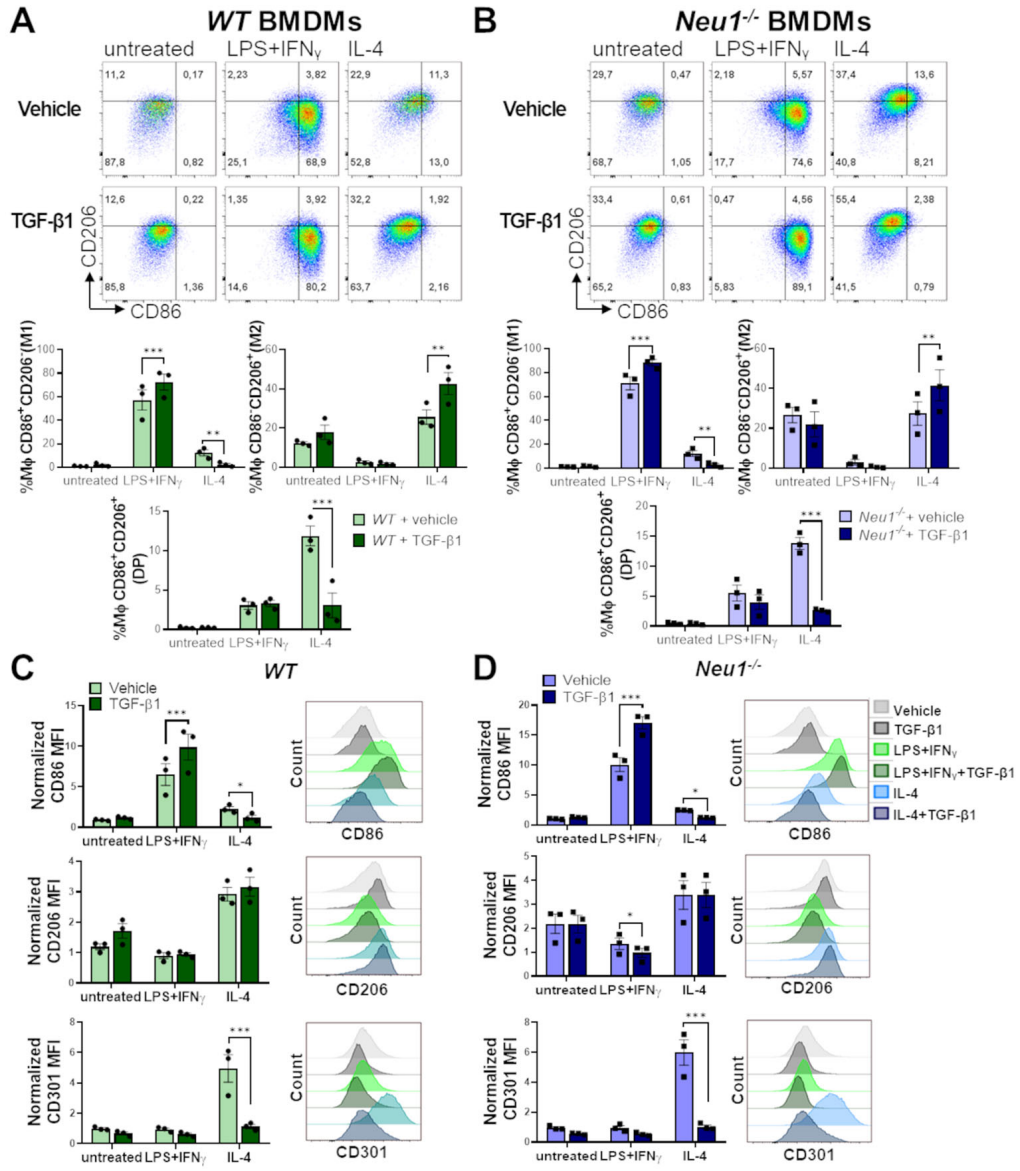
Effect of TGF-β1 on *in vitro* polarization of *WT* and *Neu*1^-/-^ BMDMs. Evaluation of TGF-β1 effect on *in vitro* polarization of *WT* **(A, C)** and *Neu*1^-/-^ BMDMs **(B, D)**. **(A, B)** (Above) Representative pseudocolor dot-plots showing CD206 and CD86 expression in *WT* **(A)** and *Neu1^-/-^* **(B)** BMDMs (CD11b^+^F4/80^+^ gated) differentiated into M1 or M2 macrophages using 100 ng/ml LPS plus 10 ng/ml IFN-γ or 100ng/ml IL-4, respectively. Additionally, cultures were simultaneously stimulated with or without TGF-β1 at 20 ng/ml. (Below) Graphs of the frequency of M1 (CD86^+^CD206^-^), M2 (CD86^-^CD206^+^) and DP (CD206^+^CD86^+^) *WT* **(A)** and *Neu*1^-/-^ **(B)** macrophages under different polarization conditions with or without TGF-β1. Bars are the mean ± SEM. *p < 0.05, **p < 0.01, ***p < 0.001 by two-way ANOVA with Bonferroni *post-hoc* test. Data from n=3 from 3 independent experiments. **(C, D)** Representative histograms and quantification of mean fluorescence intensity (MFI) of CD86, CD206 and CD301 in *WT* **(C)** and *Neu*1^-/-^ **(D)** macrophages under different polarization conditions with or without TGF-β1. MFI were normalized relative to the average of the untreated group. Bars are the mean ± SEM. *p < 0.05, **p < 0.01, ***p < 0.001 by two-way ANOVA with Bonferroni *post-hoc* test. Data from n=3 from 3 independent experiments are shown. MΦ, macrophages.

To elucidate the effect of TGF-β1 on *WT* and *Neu1^-/-^* macrophages under different polarization conditions, we analyzed M1 and M2 cell surface markers on BMDMs following M0, M1 and M2 polarization with or without TGF-β1. In resting condition, TGF-β1 did not affect M1 or M2 markers in either *WT* or *Neu1^-/-^* BMDMs (**Figures 6A, B**). However, under M1 polarization condition, TGF-β1 enhanced the frequency of M1 macrophages (**Figures 6A, B**) and increased CD86 expression in both *WT* and *Neu1^-/-^* BMDMs (**Figures 6C, D**). Additionally, TGF-β1 reduced CD206 expression only in *Neu1^-/-^* BMDMs in this condition (**Figures 6C, D**). Conversely, under M2 polarization condition, TGF-β1 increased the frequency of M2 (CD206^+^CD86^-^) cells and decreased the frequency of M1 (CD206^-^CD86^+^) and DP (CD206^+^CD86^+^) cells in both *WT* and *Neu1^-/-^* (**Figures 6A, B**), showing an overall decline in CD86 expression in both BMDMs (**Figures 6C, D**). Considering that we identified a significant number of DP macrophages (CD206^+^CD86^+^) in normal pLNs, these findings suggest that this elevated DP macrophage population in *WT* pLNs may be sustained by maintaining downregulated TGF-β1 signaling pathway. In contrast, and in line with our hypothesis, elevated TGF-β1 levels in pLNs could decrease the DP macrophages population, as observed in pLNs of *Neu1^-/-^* mice (**Figure 4**). Because CD206 expression was mostly unaffected by TGF-β1 under this condition, we evaluated another M2 marker, CD301, to analyze macrophage polarization more deeply (50, 51). Interestingly, we observed a drastic reduction in CD301 expression in both *WT* and *Neu1^-/-^* BMDMs specifically under M2 polarization condition, showing the immunomodulatory function of the cytokine (**Figures 6C, D**). Due to this dramatic TGF-β1-dependent downregulation of CD301 expression, we analyzed the CD301^+^macrophages population in the spleen and pLNs from both *WT* and *Neu1^-/-^* mice. While CD301^+^ macrophage frequency and expression in splenic macrophages were similar in both animal models (**Supplementary Figures S2A, C**), CD301^+^ macrophage percentage and expression were reduced in the pLNs of *Neu1^-/-^* mice (**Supplementary Figures S2B, D**), in line with the observed reduction of CD206 expression in LN macrophage from this mouse model. This specific reduction of the M2 marker in *Neu1^-/-^* macrophages in the TGF-β1-affected LN microenvironment, strongly supports a role for TGF-β1 in disrupting macrophage polarization in this tissue. However, since TGF-β1 did not decrease CD206 expression *in vitro* (**Figure 6D**), these results suggest that other factors in the pLN microenvironment could be contributing to the increase in the DN population of LN macrophage in *Neu1^-/-^* mice. Overall, these results emphasize the context-dependent roles of TGF-β1 and highlight that TGF-β1 might be a critical microenvironment factor that modulates M1 and M2 marker expression in LN macrophages of *Neu1^-/-^* mice.

### 3.6 *In vivo* inflammatory stimulation polarizes splenic but not LN macrophages in *Neu1^-/-^* mice

**Figure 7:**
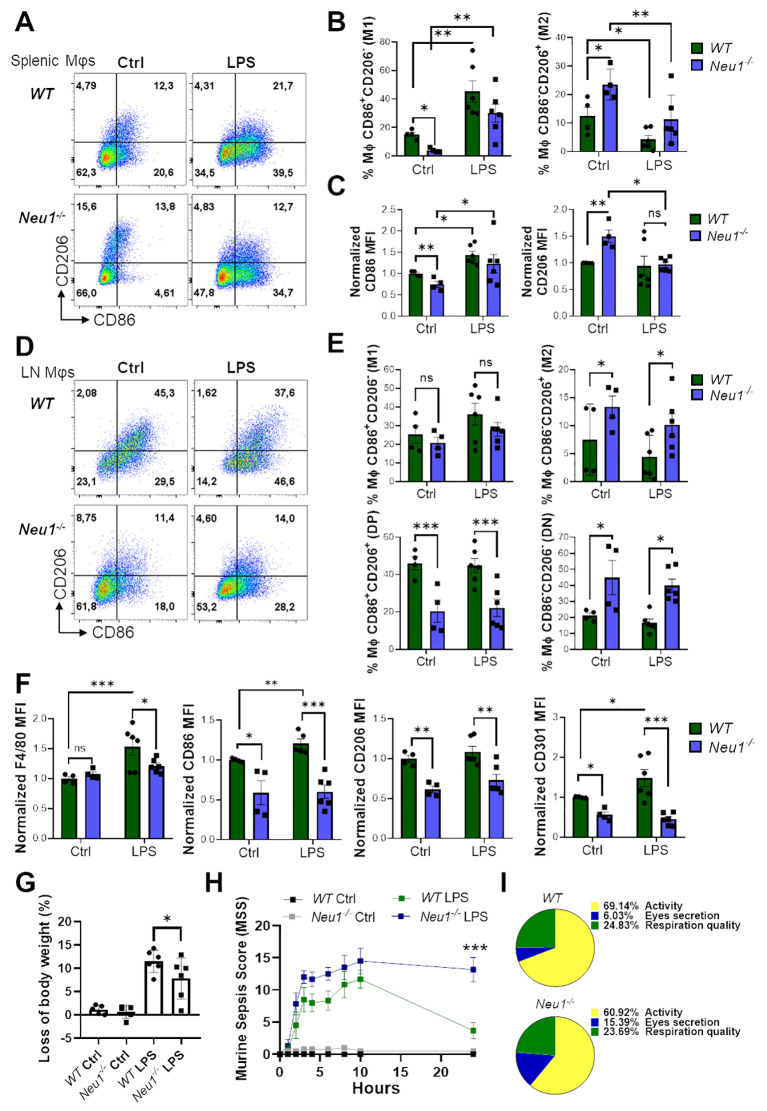
Macrophages from spleen, but not LN of *Neu1^-/-^* mice respond to *in vivo* inflammatory stimulation. **(A)** Representative pseudocolor dot-plots of CD206 and CD86 expression in F4/80^+^ splenic macrophages of *WT* and *Neu1* ^-/-^ mice after 24 hours post-injection of LPS. **(B, C)** Quantification of the frequency of M1 (CD86^+^CD206^-^ ) and M2 (CD86^-^ CD206^+^) subpopulation **(B)** and M2 (CD86^-^CD206^+^) subpopulation and relative mean fluorescence intensity (MFI) of CD86 and CD206 **(C)** of *WT* and *Neu1* ^-/-^ splenic macrophages in **(A)**. **(D)** Representative pseudocolor dot-plots of CD206 and CD86 expression in F4/80^+^ LN macrophages of *WT* and *Neu1* ^-/-^ mice after 24 hours post-injection of LPS. **(E)** Quantification of frequency of M1 (CD86^+^CD206^-^), M2 (CD86^-^CD206^+^), double-positive (DP) (CD206^+^CD86^+^) and double-negative (DN) (CD206^-^CD86^-^) *WT* and *Neu1* ^-/-^ LN macrophages showed in D. **(F)** Relative MFI of F4/80, CD86, CD206 and CD301 in *WT* and *Neu1* ^-/-^ LN macrophages in D. MFI was normalized relative to the average of *WT* mice. **(G, H)** Changes in body weight **(G)** and Murine sepsis score (MMS) **(H)** at 0, 1, 2, 3, 4, 6, 8, 10 and 24 hours after LPS injection of *WT* and *Neu1* ^-/-^ mice. **(I)** Parts of whole representation of the main indicators (activity, eye secretion, respiration) of *WT* and *Neu1* ^-/-^ mice during the whole *in vivo* inflammatory assay. Error bars represent mean ± SEM. ns: not significant, *p < 0.05, **p < 0.01, ***p < 0.001 by two-way ANOVA with Bonferroni *post-hoc* test. Data from n=4 controls and n=6 treated mice from 2 independent experiments. MΦ, macrophages.

Lastly, we investigated the effect of an *in vivo* inflammatory stimulus over the M1/M2 macrophage phenotype in SLOs of *Neu1^-/-^* mice. For this purpose, we performed an *in vivo* inflammatory assay by inducing sepsis through LPS administration in *WT* and *Neu1^-/-^* mice (31). After 24 hours, both *WT* and *Neu1^-/-^* splenic macrophages responded to the inflammatory stimuli, as evidenced by a significant increase in CD86^+^CD206^-^ (M1) frequency, along with a decrease in the CD86^-^CD206^+^ (M2) macrophage population (**Figures 7A, B**). *Neu1^-/-^* splenic macrophages were also capable of responding to the inflammatory input by increasing CD86 expression and decreasing CD206 expression to normal levels (**Figure 7C**), consistent with our findings in *Neu1^-/-^* BMDMs and *Neu1^-/-^* PMs. These results provide further evidence that the phenotype of *Neu1*-deficient macrophages can be modulated by environmental factors. Additionally, we analyzed the macrophage response to the inflammatory stimulus in the pLNs of both *WT* and *Neu1^-/-^* mice. Although we observed an increase in the CD86^+^CD206^-^ (M1) macrophage population and a decrease in the CD86^-^CD206^+^ (M2) macrophage population in *WT* mice, these changes were not statistically significant (**Figures 7D, E**). However, LN macrophages of *WT* mice significantly increase F4/80, CD86, and CD301 proteins (**Figure 7F**). In contrast, under the same inflammatory condition, LN macrophages of *Neu1^-/-^* mice did not increase the levels of these molecules (**Figure 7F**), remaining as CD86^-^CD206^-^ (DN) and CD86^-^CD206^+^ (M2) macrophages (**Figures 7D, E**), and maintained CD206 at low levels (**Figure 7F**). These results indicated that the fibrotic LN microenvironment of *Neu1^-/-^* mice has a strong immunosuppressive effect on macrophage polarization. Additionally, we monitored the weight and the murine sepsis of mice to evaluate the effect of inflammatory induction in *Neu1^-/-^* mice. Strikingly, although *Neu1^-/-^* mice appeared more resistant to sepsis because they experienced less weight loss (**Figure 7G**), they showed higher sepsis scores (**Figures 7H**, **Supplementary Figure S3**). Specifically, *Neu1^-/-^* mice were more affected in appearance, although they are usually slightly piloerected (**Supplementary Figure S3A**). The most evident difference between *Neu1^-/-^* and *WT* mice was that *Neu1^-/-^* mice had more eye secretions (**Figure 7I**, **Supplementary Figure S3D**), and after 24 hours, when symptoms subsided in *WT* mice, *Neu1^-/-^* mice still showed elevated sepsis scores (**Figure 7H**, **Supplementary Figure S3**), suggesting an abnormal innate immune response beyond the macrophage-associated immune response. In summary, our results showed that the absence of Neu1 induces macrophages to adopt an M2 phenotype, and the phenotypic features of *Neu1^-/-^* macrophages are further influenced by inflammatory and microenvironmental tissue factors, particularly TGF-β1, which govern their phenotypic characteristics.

## 4 Discussion

Macrophage polarization allows them to adapt and respond to their environment (52). Understanding macrophage polarization, which is dynamic and reversible, is crucial for improving health outcomes (53, 54). Neu1 is highly expressed in lysosomes (55) and on the cell surface of macrophages, where it cleaves sialic acids from glycoconjugates (13). Although Neu1 is not essential for macrophage differentiation, its expression and activity significantly increase during this process (13, 14), suggesting that Neu1 has effector roles in mature macrophages. In fact, Neu1 is crucial for maintaining the phagocytic capacity of macrophages (19). Previously, it was shown in cardiovascular pathologies that Neu1 downregulation promotes the arise of local anti-inflammatory cardiac monocytes/macrophages (23, 24). Our results showed that the absence of Neu1 in macrophages prompts them towards M2 polarization *in vitro*, demonstrating that the M2 phenotype is associated with genetic Neu1 deficiency in macrophages and extends beyond a phenomenon limited to cardiac macrophages (23, 24). In addition, we observed *in vivo* M1/M2 phenotypic disturbances in macrophages of *Neu1^-/-^* mice, indicating that Neu1 is critical for maintaining the normal distribution of CD206^+^ and CD86^+^ tissue-resident macrophages and their immunological roles. Moreover, a novel finding of this study is that the fibrotic microenvironment of pLNs in *Neu1^-/-^* mice also influenced M2 macrophages (CD206^+^ and CD301^+^), underscoring the relevance of fibrosis-associated factors in determining macrophage phenotypes. Among these factors, TGF-β1 demonstrated a significant role both *in vitro* and *in vivo.*

The pronounced effect of Neu1 absence on the BMDMs polarization under different polarization conditions, as well as the similar phenotypes observed in peritoneal and splenic macrophages, reinforces the idea that Neu1 modulates M1/M2 polarization. Nevertheless, this conclusion has certain limitations and should be critically considered. First, although the M1/M2 macrophage polarization is widely used to classify macrophages with a pro- or anti-inflammatory phenotype, it is essential to acknowledge the high plasticity and heterogeneity of macrophage immunophenotypes, regardless M1 and M2 are considering extremes of a spectrum of macrophage activation (52, 56). Second, most of M1/M2 macrophage polarization studies are made in BMDMs, where this classification fits well, but is less clear in tissue-resident macrophages (57, 58), where a combination of several extrinsic and intrinsic factors associated with the particularities of tissues, together with the temporal and transitory response to stimuli, commands more complex macrophage responses (52, 59). Third, certain classical polarization markers, such as CD206, play roles beyond the M1/M2 paradigm (56). For example, CD206 expression in F4/80^+^ splenic macrophages, which predominate in the red pulp of the spleen, facilitates recognition and phagocytosis of aged or damaged red blood cells, essential for the erythrocyte turnover (60, 61). Meanwhile, in other context, CD206 is involved in the phagocytosis of pathogens (62, 63). An additional limitation of our study is that, even though we observed marked *in vitro* M2 polarization of BMDMs from *Neu1^-/-^* mice after 24 hours under different conditions, using similar protocols as described previously (64, 65), the absence of a timeline study examining polarization changes at different time points limits our conclusions. This type of assay should be considered in M1/M2 polarizations assays and future analyses.

CD206 is an endocytic receptor expressed mainly in macrophages that binds and internalizes a variety of endogenous and pathogenic glycans (66, 67), which is susceptible to regulation by sialylation. CD206 contains extracellular N-glycosylation sites where terminal sialic acids are necessary to bind and internalize mannose-containing CD206 structures (68, 69). As during macrophages differentiation or in an inflammatory context, Neu1 translocates to the cell surface of macrophages (13) to desialylate surface proteins and receptors (21, 22, 70), we infer that Neu1 may regulate CD206 function or location at post-translational level, especially considering that we did not observe changes in CD206 gene expression in *Neu1^-/-^* BMDMs after M1/M2 stimuli, yet we observed CD206 surface accumulation. Regarding location, the absence of terminal extracellular sialic acid does not affect the plasma membrane location or the endocytic activity of mannose-bearing glycans by the CD206 receptor (71). However, as Neu1 also has a role in intracellular trafficking modulation (72), it is possible that Neu1 regulates CD206 location by modulating cellular trafficking, explaining the elevated levels of CD206 at the cell surface of *Neu1*-deficient macrophages. On the other hand, CD206 has plenty of roles in homeostasis, such as in collagen turnover tissues (73). Sialylated CD206 cannot bind collagen, which depends on CD206 multimerization, a process that has only been observed when the receptor is not sialylated (69, 74). Therefore, it will be reasonable that CD206 in the absence of Neu1, and probably in a permanent hypersialylated status, could be performing abnormal uptake of tissue collagens, leading to an imbalance in collagen turnover in tissues (75). The altered function of CD206 in *Neu1^-/-^* mice could be an additional axis, besides the previously described exacerbated exocytosis of fibrotic factors intrinsic to *Neu1^-/-^* fibroblasts, contributing to the generalized fibrosis observed in many organs (42), as well as in pLNs of *Neu1^-/-^* mice. In contrast, despite the severe systemic fibrosis in *Neu1^-/-^* mice, we did not find elevated levels of TGF-β in the spleens of *Neu1^-/^* ^-^ mice. Although the tissue peculiarities need further analysis, it could be a consequence of the particular microenvironment of enlarged spleens (splenomegaly) with elevated extramedullary hematopoiesis (76).

Fibrosis in LNs has been described in lymphoedema (77) and associated with kidney fibrosis (78), where FRCs play a determining role in fibrosis progression (79). Additionally, fibrosis has been observed in metastatic LNs, where it correlates with poor patient prognosis (80, 81), highlighting the importance of understanding the mechanisms associated with this process. Moreover, a prior study linked LN fibrosis to weakened immunological responses to vaccines (82). Consistent with this, M1/M2 macrophage polarization imbalance in the fibrotic LN of *Neu1*-deficient mice, which was not altered even by *in vivo* inflammatory stimulation, could be closely associated with the unsuccessful immune reactivity to vaccinations and should be considered in immunization schedules. Furthermore, we also found an enrichment of DP (CD86^+^CD206^+^) macrophages in the pLNs of *WT* mice. DP macrophages have been described in tumors (83, 84), where they have prognostic value, associated with tumor aggressiveness but their inflammatory function is unclear, and it is uncertain whether they correspond to a transitory, intermediary, or well-defined phenotype. In the case of the F4/80^+^CD86^+^CD206^+^ homeostatic macrophages that we found in the pLNs, we speculated that the double expression of CD86 and CD206 could be associated with their function in phagocytosis as well as with antigenic presentation, since these functions have been previously associated to F4/80^+^ LN macrophages from the medullary region of LNs (40, 56), but this hypothesis needs to be further evaluated. In contrast, DP macrophages were strongly underrepresented in the pLNs of *Neu1^-/-^* mice, associated with TGF-β1 overexpression and FRCs activation. Interestingly, the DP macrophage population decreased following *in vitro* treatment with TGF-β1 in the presence of IL-4, demonstrating that TGF-β1 can influence DP macrophages under specific conditions, as observed in the pLNs of *Neu*1^-/-^ mice and suggesting that IL-4 may also be a dysregulated microenvironmental factor in the fibrotic pLNs of these mice.

Macrophages are known to produce TGF-β1 (49, 85). Our results did not show any difference in TGF-β1 expression between *WT* and *Neu1* ^-/-^ macrophages *in vitro*, suggesting that *Neu1^-/-^* macrophages may not be involved in the increased TGF-β1 levels observed in the pLNs of *Neu1^-/-^* mice. This is consistent with evidence showing that macrophage depletion has minimal impact on TGF-β1-dependent fibrosis (86). The role of Neu1 in regulating the secretory pathways of TGF-β1, as well as the activation of TGF-β1 or its receptors, needs further investigation. Previously, it has been shown that myofibroblasts from *Neu1^-/-^* mice exhibit higher exocytosis of TGF-β1 (42), this evidence, along with the observation of more activated features and complexity of FRCs in *Neu1^-/-^* mice, our results strongly suggest that FRCs in the pLNs of *Neu1^-/-^* mice could be involved in producing higher levels of TGF-β1 and ECM components. FRCs maintain the LN macrophages parenchymal niche (87), therefore, the elevated number of FRCs in the LNs of *Neu1* ^-/-^ mice may be responsible for the increased number of LN macrophages observed in *Neu1* ^-/-^ mice. In addition, the close association of FRCs with macrophages and other immune cells (87, 88) could also be facilitating the immunosuppression of LN macrophages and altering the LN microenvironment in *Neu1* ^-/-^ mice. Moreover, the potential contributions of other cells, such as lymphatic endothelial cells (89), epithelial cells or T cells (85, 90), to the elevated TGF-β1 levels and fibrosis in the pLNs of *Neu1* ^-/-^ mice cannot be excluded. Additionally, FRCs are crucial in organizing LN structure during homeostasis and inflammation, guiding immune responses, and forming the foundation for immune cell organization (88, 91), with Sca1 and PDPN proteins playing key roles (45, 46, 92). The increased expression of Sca1 and PDPN in FRCs from *Neu1* ^-/-^ mice suggests that these FRCs are actively modifying the LN microarchitecture in *Neu1* ^-/-^ mice, although further research is necessary to confirm these observations.

It is well known that TGF-β is strongly associated with M2 macrophages because TGF-β induces M2 macrophage polarization and is the major pro-fibrotic cytokine that induces fibroblasts to produce extracellular matrix (ECM) (28, 93). However, the effect of TGF-β on macrophages appears to be associated with macrophage subtypes. Thus, while TGF-β induces M2 polarization and CD206 expression in THP1 human macrophages (28), in alveolar macrophages, TGF-β decreases CD206 expression (94). Additionally, long exposure to TGF-β1 prevented microglia from switching from the M1 to M2 phenotype, impairing their capacity to develop a resolving anti-inflammatory phenotype (95). From our results, we observed that LN macrophages were responding to chronic TGF-β1 exposure in the pLNs of *Neu1^-/-^* mice, leading to an elevated M2 and DN macrophage population and globally downregulated CD206 and CD301 levels, similar to what was described in alveolar macrophages and microglia, and this response was sustained even after inflammatory induction in *Neu1^-/-^* mice. These results could provide an explanation about why the therapeutic inhibition of Neu1 has been shown to be particularly protective in pulmonary fibrosis (96, 97), whereas constitutive or phagocyte-specific Neu1 depletion increased renal fibrosis (72), liver, or skeletal muscle fibrosis (42). Nevertheless, the role of Neu1 in idiopathic pulmonary fibrosis (IPF) is contradictory because one study showed Neu1 upregulation in lung samples from IPF patients (96), while another study showed Neu1 downregulation (42). Furthermore, we observed that TGF-β1 effects on macrophage polarization depend on the immunological context. TGF-β1 showed a dual role, increasing M1 markers under LPS-dependent inflammation and decreasing them under IL-4-dependent immune response, independent of the genetic status of Neu1. The microarchitecture disturbance of the pLNs in *Neu1^-/-^* mice could support several signals implicated in downregulating macrophage polarization. Additionally, the fact that SLOs from *Neu1^-/-^* mice displayed more macrophages, meanwhile in the peritoneum, they had fewer macrophages, could indicate alterations in macrophage homing regulation. In fact, Neu1 deficiency is associated with ECM remodeling in the bone niche, which alters the retention of hematological progenitors in the bone marrow (11).

Despite the immunosuppressive LN microenvironment and the presence of M2 macrophages at the peritoneal cavity and spleen of *Neu1^-/^* ^-^ mice, these mice were not protected from sepsis and exhibited more severe symptoms, highlighting the role of Neu1 in the innate immune system and immune physical barriers in the inflammatory response. For instance, among other mechanisms, surface sialylation modulates neutrophil viability (98), and Neu1 deficiency elevates neutrophil protease exocytosis in the bone marrow niche (11). Additionally, Neu1 is expressed in the airway epithelia, especially within the brush border, where it can regulate the Epidermal growth factor receptor (EGFR) and Mucin1 (99), which serve as lubricants and physical barriers to protect cells from damage or infections, but are also associated with chronic inflammation (100).

In summary, we showed here that Neu1 deficiency leads to an abnormal M1/M2 macrophages phenotype *in vivo* and under M1/M2 polarizing conditions, which could be affected by the microenvironment. The fact that external microenvironment factors can modulate the phenotype of *Neu1*-deficient macrophages opens a therapeutic window for treating inflammatory and fibrotic conditions as well as genetic diseases such as sialidosis, an autosomal recessive disorder caused by mutations in the *Neu1* gene (101) or galactosialidosis, a combined deficiency of Neu1 and cathepsin A (102). Interestingly, although various defects in macrophage populations have been described in *Neu1* ^-/-^ mice (11, 19), and our study has now added insights into M1/M2 polarization, still the status of macrophages in sialidosis patients is currently unknown. Further studies are necessary to specifically evaluate the phenotype and function of macrophages in sialidosis patients to corroborate and expand upon our findings.

## Data Availability

The original contributions presented in the study are included in the article/**Supplementary Material**. Further inquiries can be directed to the corresponding authors.
