# Supplementary material for: Neu1 deficiency and fibrotic lymph node microenvironment lead to imbalance in M1/M2 macrophage polarization
Source: Front Immunol. 2024 Sep 13;15:1462853. doi: 10.3389/fimmu.2024.1462853 (PMC11427323; doi:10.3389/fimmu.2024.1462853)
Supplement: Supplementary file 1 [file DataSheet1.pdf]

Supplementary Material

1 Supplementary Figures

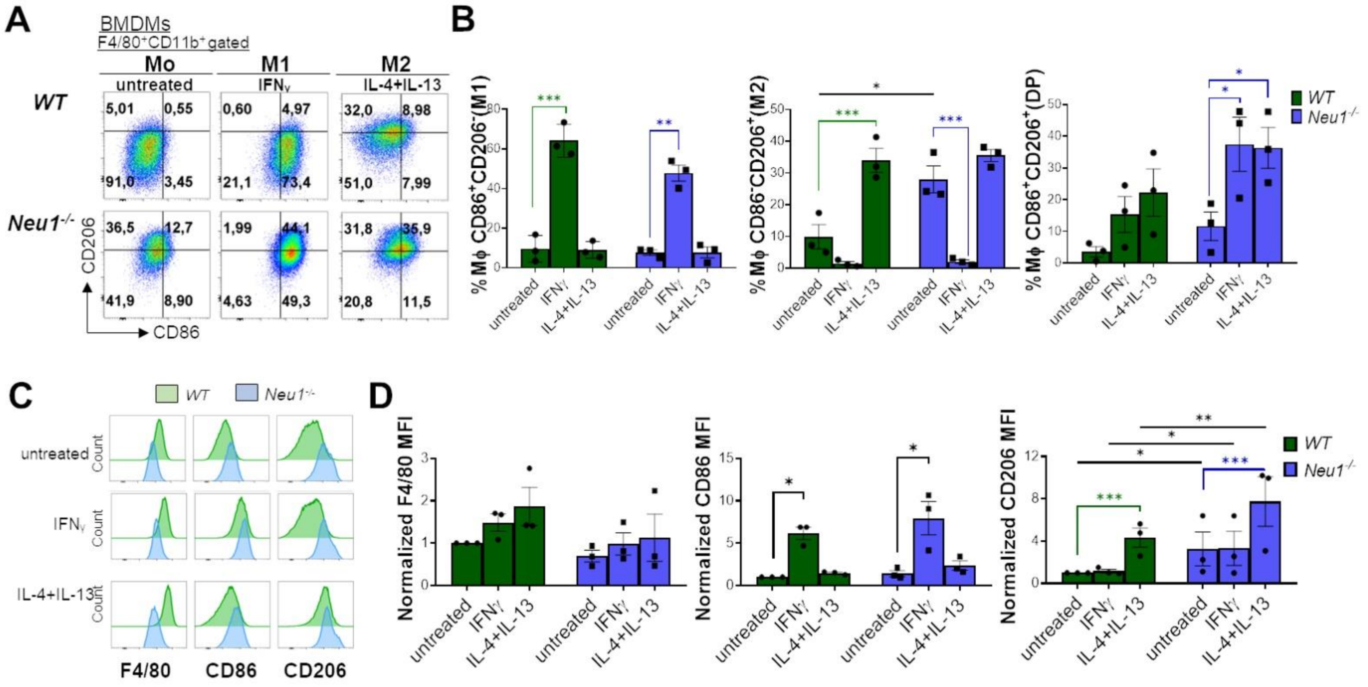

Supplementary Figure 1. Analysis of *WT* and *Neu1*$^{-/-}$ BMDMs phenotypes after *in vitro* M1 and M2 polarization. A. Representative pseudocolor dot-plots showing CD206 and CD86 expression in *WT* and *Neu1*$^{-/-}$ BMDMs after 24 hours of *in vitro* polarization with IFN-γ (100 ng/ml), IL-4 plus IL-13 (20 ng/ml and 10 ng/ml, respectively) and medium (untreated). **B.** Quantification of the frequencies of M1 ($CD86^{+}CD206^{-}$), M2 ($CD86^{-}CD206^{+}$) and double-positive (DP) ($CD206^{+}CD86^{+}$) macrophages in *WT* and *Neu1*$^{-/-}$ BMDMs after polarization. **C-D**. Representative histograms and graphs of F4/80, CD86 and CD206 expression in *WT* (green) and *Neu1*$^{-/-}$ (blue) BMDMs after *in vitro* polarization. For quantification, mean fluorescence intensity (MFI) was normalized to the average of the untreated *WT* group. Bars represent mean ± SEM (n=3). *p < 0.05, **p < 0.01, ***p < 0.001 by two-way ANOVA with Bonferroni post-hoc test.

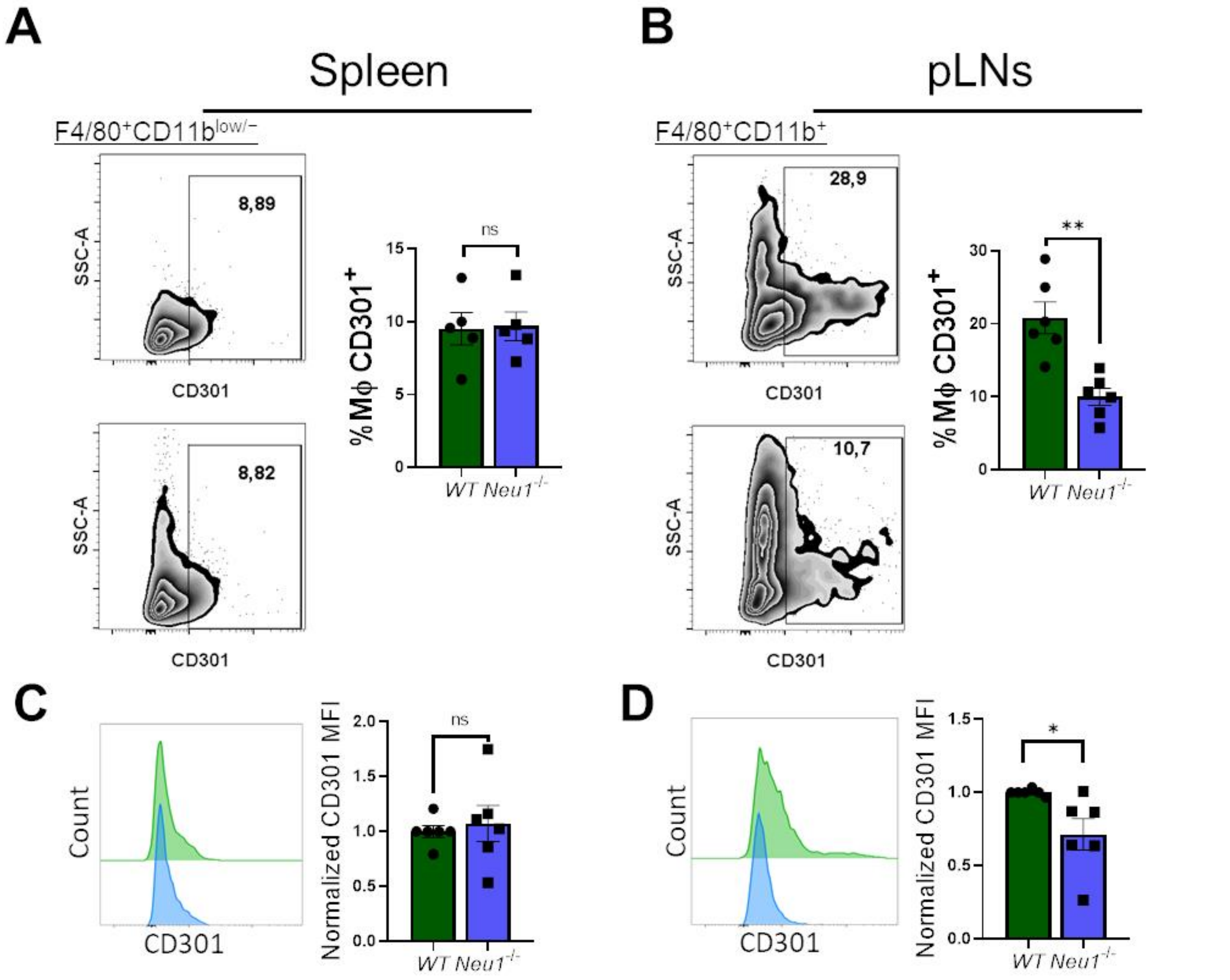

Supplementary Figure 2. Evaluation of CD301 expression in splenic and LN macrophages of WT and *Neu1*$^{-/-}$ mice. A-B. Representative zebra plots and quantification of CD301^{+} splenic (**A**) and LN (**B**) macrophages from *WT* and *Neu1*$^{-/-}$ mice. **C-D** Representative histograms and relative expression of CD301 in splenic (**C**) and LN (**D**) macrophages (CD11b^{+} F4/80^{+}) from *WT* and *Neu1*$^{-/-}$ splenic macrophages. Mean fluorescence intensity (MFI) was normalized relative to the average of *WT* mice. Bars represent mean ± SEM. ns: not significant, *p < 0.05, **p < 0.01 by unpaired t-test. n=6 from 3 independent experiments.

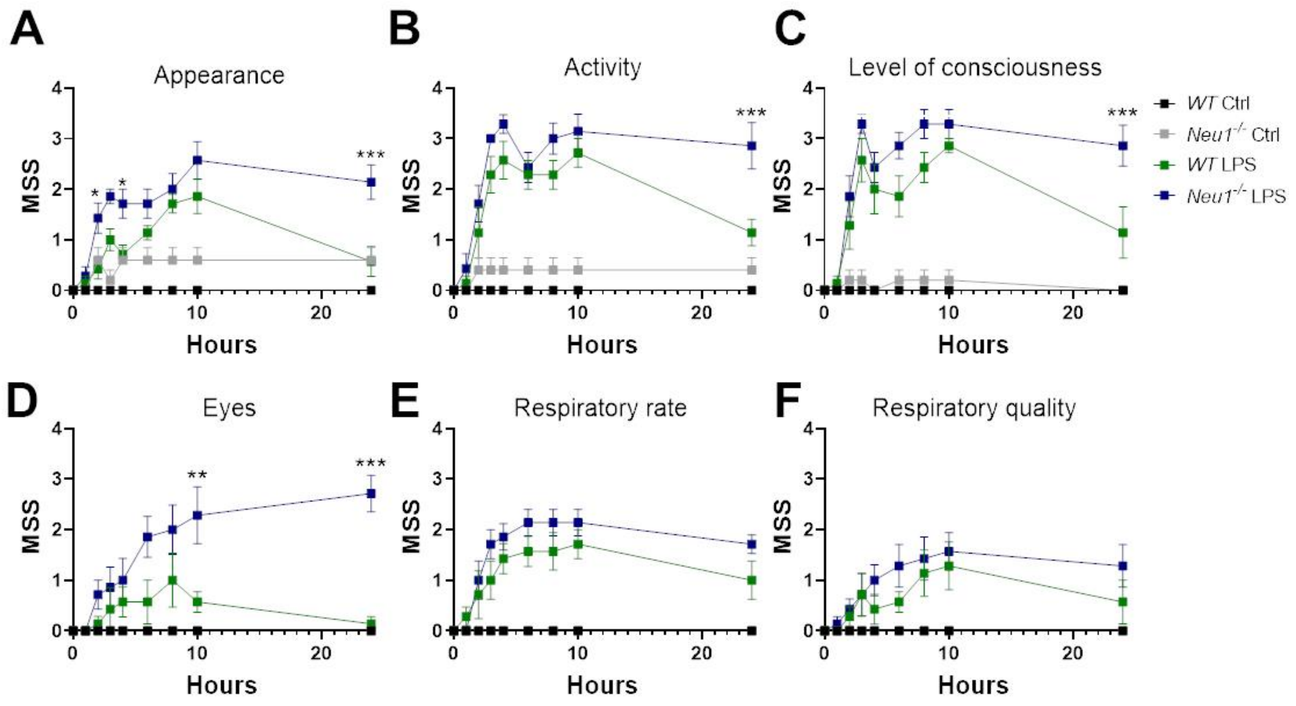

Supplementary Figure 3. Individualized murine sepsis score (MSS) indicators during the *in vivo* inflammatory assay of *WT* and *Neu1*$^{-/-}$ mice. Individualized MMS values for appearance (**A**), activity (**B**), level of consciousness (**C**), eyes secretion (**D**), respiration rate (**E)** and respiration quality (**F)** of *WT* and *Neu1*$^{-/-}$ mice during the *in vivo* inflammatory assay at 0, 1, 2, 3, 4, 6, 8, 10 and 24 hours. Error bars represent mean ± SEM. ns: not significant, *p < 0.05, **p < 0.01, ***p < 0.001 by two-way ANOVA with Bonferroni post-hoc test. Data from n=4 controls and n=6 treated mice from 2 independent experiments.
